# Toughness measures in solid-state composites of ultra-high molecular weight polyethylene

**DOI:** 10.1016/j.jmbbm.2025.107136

**Published:** 2025-07-12

**Authors:** Peder C. Solberg, Igor Tsukrov, Douglas W. Van Citters

**Affiliations:** a Dartmouth College, Hanover, NH, 03755, USA; b University of New Hampshire, Durham, NH, 03824, USA

**Keywords:** UHMWPE, Arthroplasty, Composites, Fatigue, Toughness, Nanocomposites, Fracture

## Abstract

Solid-state composites of ultra-high molecular weight polyethylene (UHMWPE) are increasingly being investigated for various therapeutic and sensing use cases in arthroplasty. Due to its extremely high melt viscosity, composites of UHMWPE exhibit a much more distinct phase segregation than lower-viscosity polymer composites. This phase segregation may influence UHMWPE behavior in a different way than microstructural factors studied previously (e.g. crosslinking). The purpose of this study was to quantify UHMWPE nanocomposites’ resistance to failure via several distinct measures, across a range of carbon black (CB) filler loadings. Results showed that tensile and impact toughness followed similar trends: both maintained or increased in magnitude over neat controls at low filler concentration, then decreased at higher filler contents. On the other hand, fatigue crack propagation resistance (CPR) demonstrated similar behavior to tensile and impact toughness at lower concentrations but diverged at higher concentrations. From 2.5 wt% CB to the highest concentration tested (10 wt% CB), fatigue properties improved—unlike tensile or impact toughness. The observed transitions in macroscopic behavior with increasing filler content coincided with microstructural phenomena observed via scanning electron microscopy (SEM) and fracture surface imaging. These phenomena included a transition from transgranular fracture to intergranular fracture, the onset of complete granule coating, and the formation of intergranular voids. This work demonstrates that tensile and impact toughness are not necessarily indicative of fatigue CPR in these materials. Broadly, the findings presented in this study motivate further investigation of structure-property relationships for phase-segregated polymer composites and demonstrate promise for their use in high-load scenarios.

## 1. Introduction

Modern joint replacement surgery (arthroplasty) has been prevalent for over 60 years and is generally a very high value way to improve the lives of patients with osteoarthritis. As a result of this success, arthroplasties have become some of the most commonplace elective procedures completed in operating rooms today, with approximately 790,000 knee replacements, 540,000 hip replacements, and 110,000 shoulder replacements completed annually in the US with volumes expected to continue growing (Ray, 2024; Wagner et al., 2020; Singh et al., 2019). However, despite their success, many challenges remain in arthroplasty. For example, loosening, instability, and infection have persistently remained among the top reasons for implant failure leading to revision surgery (Lum et al., 2018). Furthermore, with more joint replacements being performed, especially in younger patients, revision surgery will continue to place a significant burden on the healthcare system. The lifetime risk of revision for knee arthroplasty patients age 50–54 is 35%, compared to 5% for patients aged 70 and older (Schreurs and Hannink, 2017). With high costs for revision surgeries and degraded patient quality of life following implant failures, anything that can be done to prevent these failures or improve outcomes will yield economic and quality of life benefits to those patients and society as a whole (Schwartz et al., 2021).

In the past 10–15 years, researchers and some device manufacturers have become interested in a variety of solid-state composites for addressing challenges in arthroplasty (Asik et al., 2025; Chen et al., 2012; Currier et al., 2018; Dong and Qi, 2015; Gil et al., 2023; Inverardi et al., 2025; Lahiri et al., 2012, 2014; Lekkala et al., 2024b; Narayan, 2016; Suhardi et al., 2017; Tai et al., 2012). These materials are distinct from early solid-state composites used in arthroplasty, which integrated carbon fibers in UHMWPE but failed due to inadequate carbon-polymer adhesion and oxidation leading to large abrasive wear rates and fracture for some implants (Kurtz, 2009). In contrast, new generations show promise as they use powder-based fillers which achieve better filler integration into bulk materials. Specifically, fillers with small particle size, such as carbon black nanoparticles, have a much larger surface area per mass available for interacting with surrounding materials than micro-sized particle fillers. This large surface area allows for more interaction with the bulk materials if agglomerations can be avoided (Fu et al., 2008, 2019). Thus, polymer composites with finer particle size are generally known to exhibit better mechanical properties (Asik et al., 2025; Senatov et al., 2014). Fine particle size is also known to result in favorable elution properties in some therapeutic forms of solid-state composites, providing broad motivation for the study of mechanical properties in solid-state composites of UHMWPE with nanoscale filler particles (Asik et al., 2025).

Due to the high viscosity of UHMWPE, the microstructure of its solid-state composites exhibits a much more distinct phase segregation than lower-viscosity polymer composites. Prior work in our labs with carbon-loaded UHMWPE composites showed that the carbon black phase and the polymer phase only interact around the outside of polymer granules. This creates filler-rich granule boundary regions, which contain an estimated 30–45% carbon black filler by volume (Buklovskiy et al., 2024). The effect of this microstructure on these materials’ ability to resist mechanical failure in the body is not adequately understood and thus is the subject of this study.

A variety of solid-state filler powders have been integrated into UHMWPE in recent years, such as antibiotics to treat prosthetic joint infection (PJI), analgesics to help prevent PJI, particulate antioxidants to prevent oxidative degradation of polymer bearings, and conductive nanoparticles to create electrically-conductive bearing domains (Lekkala et al., 2024a, 2024b; Bracco and Oral, 2011; Suhardi et al., 2017; Gil et al., 2023; Inverardi et al., 2025; Asik et al., 2025; Currier et al., 2018; Clark et al., 2006). Several of these composites are already ushering in the next generation of orthopedic devices by addressing key challenges in arthroplasty. While the present study seeks to elucidate the mechanical behavior of nanocomposites of UHMWPE generally, the specific composites studied herein may be useful for novel types of orthopedic bearings. For example, integration of electrically conductive carbon black domains into UHMWPE bearings could be useful for applications including electrically stimulated biofilm disruption or integration of sensing systems into bearing components (Clark et al., 2006; Ehrensberger et al., 2015).

With a range of loading scenarios expected in orthopedic bearings made with solid-sate composites of UHMWPE, we seek to better understand the tensile toughness, Izod impact toughness, and fatigue crack propagation resistance of carbon black-UHMWPE bearings in the context of microstructural features. These three different measures of toughness demonstrate the ability for these candidate bearing materials to resist mechanical failure in low strain rate, high strain rate, and cyclic crack propagation scenarios, respectively. Historically, they have been used to understand other new forms of UHMWPE, such as crosslinked materials, as those materials tended to have reduced properties across all three measures as a tradeoff for improved wear behavior (Atwood et al., 2011; Baker et al., 2003; Gencur et al., 2006; Huot et al., 2011; Morrison and Jani, 2009). One recent study (Inverardi et al., 2025) reported on tensile, impact, and fatigue properties for drug-loaded UHMWPE composites and discussed the effects of certain kinds of filler morphology on mechanical properties. However, it did not seek to comprehensively investigate the effect of filler concentration across all three measurement categories, nor did it focus exclusively on nano-sized fillers, as we will here.

Studies of carbon-UHMWPE composites have investigated the effect of nano-sized fillers such as carbon nanotubes and reduced graphene oxide, reporting mechanical properties such as tensile strength and impact resistance, but have not included studies of multiple toughness measures for these materials. Nonetheless, such studies suggest that toughness and properties contributing to toughness (e.g, tensile strength) will tend to change with the addition of nano-sized carbon filler particles. Generally, increasing filler concentration leads to decreases in these mechanical properties, but occasionally improvements have been observed at certain low filler concentrations (Eun et al., 2022; Mierczynska et al., 2007; Rama Sreekanth and Kanagaraj, 2013). The extent to which solid-state fillers affect these three toughness measures across a range of nano-sized filler concentrations is not adequately understood, motivating the present study.

With this background in mind, the objective of this study is to understand (1) how increases in filler concentration affect tensile toughness, impact toughness, and fatigue crack propagation resistance, and (2) to observe and report microstructural phenomena associated with the observed increases or decreases in toughness measures at (a) high, (b) low, and (c) moderate concentrations. Objective 2 will be accomplished via fracture surface imaging and scanning electron microscopy (SEM) to determine potential micro and nano-structural reasons for the observed toughness behaviors. This study will thus provide insights into the behavior of both the carbon black nanocomposites of UHMWPE being studied here and other solid-state composites of UHMWPE subjected to various loading scenarios in applications of interest. In doing so, with this scoping study we aim to lay the foundation for future work to investigate structure-property relationships for these materials in more depth.

## 2. Materials and methods

### 2.1. Material fabrication

**Fig. 1.**
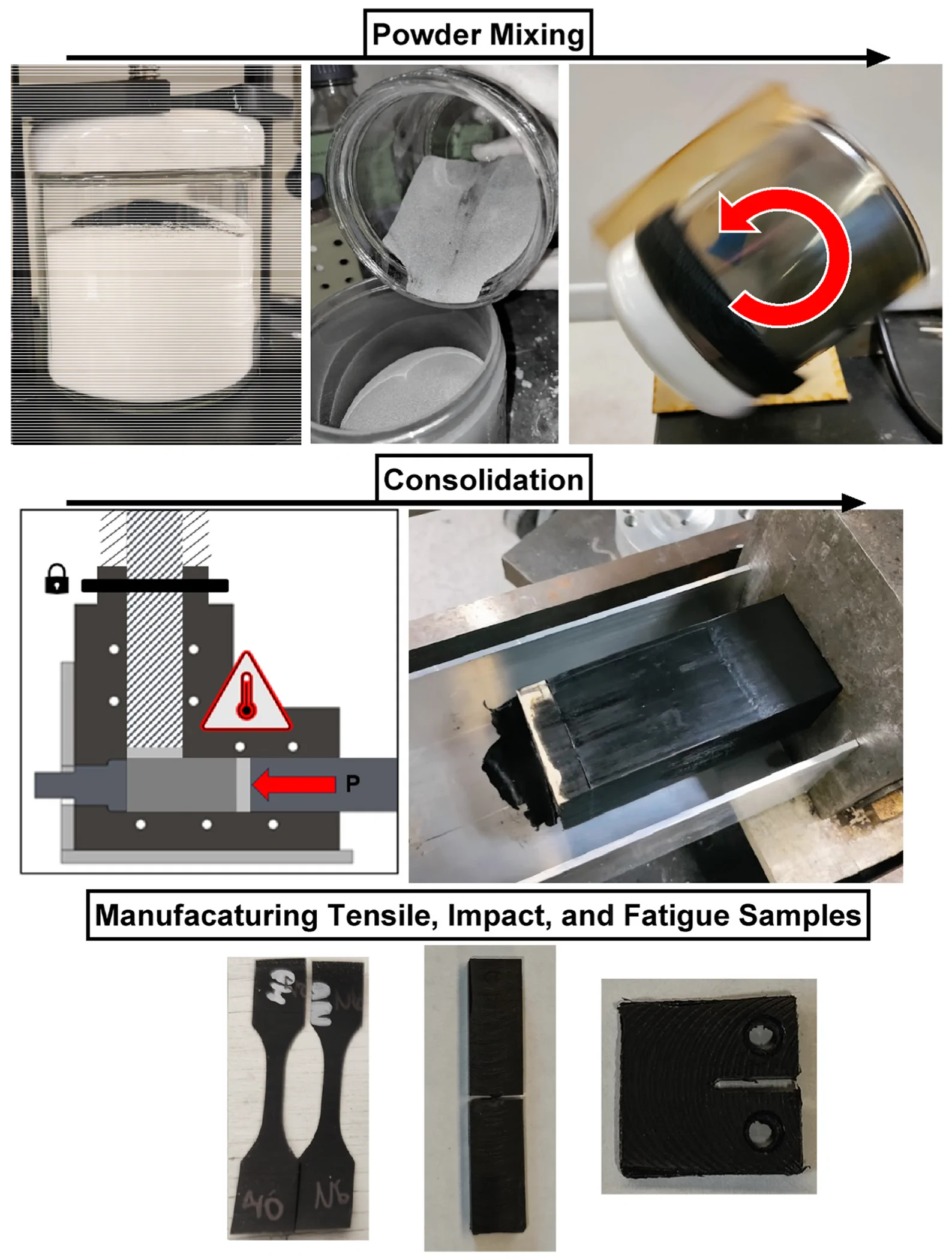
Manufacturing processes completed to produce test samples for this study.

Material treatments were selected to represent materials across a range of filler concentrations: 0, 0.5, 1, 2.5, 5, and 10 wt% carbon black in UHMWPE. The high end of this range was set to be higher than many other past studies of carbon-UHMWPE nanocomposites (Puértolas and Kurtz, 2016), but not so high that the billets experienced thermal cracking during their post-consolidation cooling process, according to preliminary testing. Several intermediate concentrations were added for tensile analyses only to provide more information about the composites’ behavior near the onset of electrical continuity (0.25 wt% and 0.75 wt %), and at a moderately-high concentration (7.5 wt%). To prepare bulk materials, UHMWPE powder (Celanese GUR 1020, average molecular weight 3.5 million g/mol, typical particle size 100–200 μm) and pigment carbon black (PCB) powder (Asbury 5388 Pigment Black, primary particle size 19 nm) were weighed for the desired filler concentrations and placed in a thick-walled glass mixing vessel. Powders were acoustically mixed at 100 G acceleration for 3 min at 60 Hz in a Resodyn LabRAM I resonant acoustic mixer to achieve dispersive mixing of the powders (Resodyn, Butte, MT). The mixing vessels were filled to two-thirds of capacity to allow space for energetic mixing. Acoustic mixing was followed by lower-velocity mixing wherein the cylindrical mixing vessel was mounted to a motorized plate and tumbled end-over-end for 2 min at 40 RPM to achieve a higher level of distributive mixing of the separate powder phases (see Fig. 1). Two 140 g batches were used to make each billet, as the volume of the mixing vessels was limited. Neat samples were also acoustically mixed to ensure consistency with the composites.

Powders were then compression molded in the horizontal portion of a steel die described previously (Reinitz et al., 2016) at 14 MPa and 175 °C for 2.5 h. This time and temperature are similar to those used for consolidation by Favreau (Favreau, 2022; Favreau et al., 2022) and Reinitz (Reinitz et al., 2016), who produced billets of similar geometry to those considered in this work. The consolidation pressure was selected to be in the 8–20 MPa range common for processing of UHMWPE composites (Clark et al., 2006; Gil et al., 2020; Gupta et al., 2013; Mallick et al., 2020). Initial heating took 20–30 min, during which time the horizontal die plunger was actuated occasionally to push out air pockets and decrease powder volume until a fully dense mass was obtained and held at temperature for the remaining ~2 h. Consolidated billets with approximate dimensions 15x5x5 cm were removed immediately via an ejection plunger and allowed to cool overnight at room temperature before being processed further.

Tensile, impact, and fatigue test samples were manufactured from the consolidated billets, according to specifications and guidance referenced in Table 1. While compression molding of polymers is generally known to produce isotropic samples (per ASTM D4703-24, section 4.1), slight anisotropies might occur in neat UHMWPE materials such that the location and orientation of samples can matter, even when compression molded (ASTM F648-21, A1.4.1). Thus, care was taken to ensure samples were extracted from consistent regions and with consistent orientation across the material treatments. Tensile and fatigue samples were taken from matching regions on opposite sides of the front of the billet, while impact samples were taken from the rear of the billet. A 3 mm rind around the outside of the billet was always avoided. For all three specimen types, samples were machined such that the direction of tensile loading in the deformed volume of interest was parallel to the direction of compressive loading during consolidation. For impact and fatigue samples, this meant that the direction of crack propagation was perpendicular to the direction of compression molding as specified in ASTM F648.

### 2.2. Mechanical testing - tensile

Tensile testing was completed on an Instron 68SC-2 electromechanical load frame (Instron, Norwood, MA) using the modified ASTM D638 Type V samples described in Table 1 and a 500 N load cell. Samples were marked with two small white tape marks placed 10 mm apart so that sample strain could be tracked in the gauge region with the Instron video extensometer. All samples were strained to failure at a crosshead speed of 25.4 mm/min, equating to a strain rate of approximately 100% per minute. The area under the stress-strain curve was computed to report tensile toughness (Roylance, 2020). Other tensile properties reported include strain at break (ductility) and ultimate tensile stress (UTS). The resulting data were analyzed with Brown-Forsythe and Welch ANOVA followed by Dunnett’s multiple comparisons tests using GraphPad Prism version 10.2.2 for Windows (GraphPad Software, Boston, Massachusetts USA). Materials treatments (different concentrations of carbon black) were treated as categorical variables, rather than continuous numerical variables, for the sake of statistical comparison between groups. A p-value <0.05 was considered statistically significant. For each material treatment, 15 samples were analyzed. Additional samples were produced to account for occasional operational failure of the data collection system (e.g., lost marks) and were only used as necessary to maintain statistical power.

### 2.3. Mechanical testing - impact

Impact testing was completed on an Izod impact testing machine (CEAST Resil Impactor, Type 6957, Instron, Norwood, MA) as described previously (Huot et al., 2011) and in accordance with ASTM F648. Briefly, a 5.5 J hammer was used to strike the sample, and the energy absorbed by the specimen and hammer was recorded. This value was corrected for losses due to air resistance and friction by subtracting the pendulum energy loss recorded during an empty run. To get the final energy, this corrected energy value was divided by the cross-sectional area subjected to strain and measured via calibrated optical microscope images of the fractured sample surfaces. Statistical testing utilized Dunnet’s T3 multiple comparisons tests with *p* < 0.05 considered significant.

### 2.4. Mechanical testing - fatigue

For fatigue testing, compact test specimens with outer dimensions per Baker et al. (2003) were subjected to defect-tolerant fatigue testing based on ASTM E647 and using the specific methodology employed by Atwood and colleagues, wherein loads were incrementally increased at each segment of testing (Atwood et al., 2011). To maintain a mix of plane stress and plane strain conditions expected to be present in orthopedic bearing features, samples in this study did not contain a machined groove at the side of the crack plane. However, samples were checked following each test to ensure that the crack had grown along the plane normal to the load application direction. A sharp razor blade and custom jig were used to introduce a sharp crack into the compact test specimen notch as described previously (Atwood et al., 2011). A load ratio R = 0.1 was used with a sinusoidal tensile loading waveform applied at 5 Hz by an Instron 8501 servohydraulic load frame and an Instron 8800 control system (Instron, Norwood, MA). An air jet was pointed at the crack tip to counter hysteretic heating of the samples. Loading segments were 10,000 cycles long, with maximum load increased by 10 N from one segment to the next (e.g. 9–90 N, then 10–100 N, etc.). Five samples were tested at each of six concentrations as described in Table 1. Samples were run in a randomized order to mitigate the effects of any unaccounted systematic variables, e.g. changes in room temperature.

**Fig. 2.**
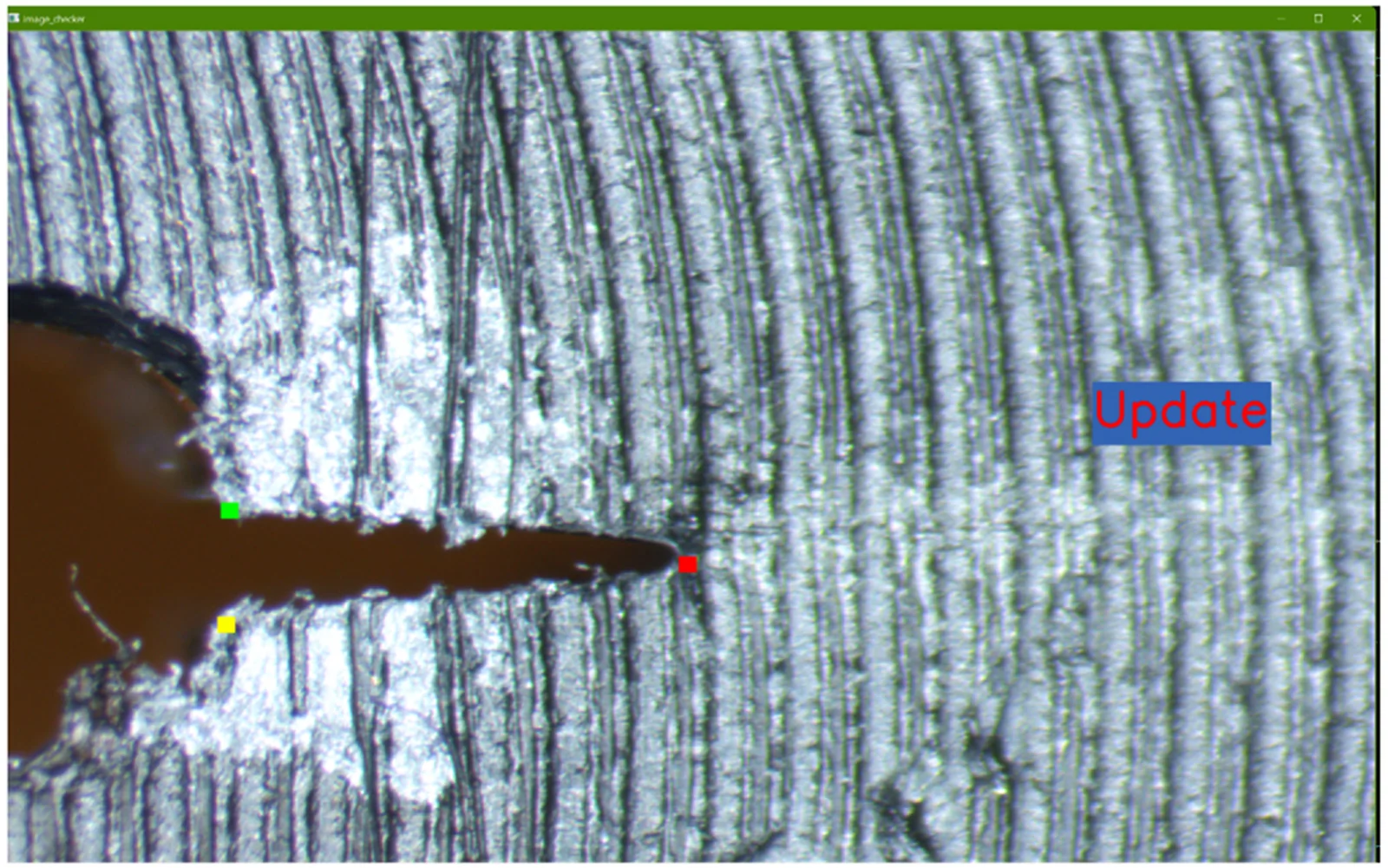
Custom Python GUI used to measure length of razor crack. Red mark - farthest rightward point of opening, indicating crack tip. Yellow and green - corners used as fiducial markers with known location relative to the location of load application. As the crack grows, the assigned position of the red marker moves rightward relative to the green and yellow markers. (For interpretation of the references to color in this figure legend, the reader is referred to the Web version of this article.)

To record crack propagation at regular intervals, images were taken of both sides of the crack tip using separate 16 MP microscope cameras with 0.5x reduction lenses (SwiftCam SC1603, Swift Microscopes, China), mounted to an Amscope boom microscope with the objective set to 4.5× and 0.5x reduction lenses installed. Resolution of this system was 1.2 μm/pixel. Calibration images were taken after system setup and again either before or after each test to ensure consistency. Total crack length was assessed for each image by measuring the horizontal distance from the start of the razor crack to the crack tip and adding the machined distance from the load application plane to the start of the razor crack, to get the total crack length as defined by ASTM E647. A custom Python user interface was used to assign markers and determine crack length in pixels as shown in Fig. 2. Calibrations were applied during the subsequent analysis to convert from pixel units to length units.

Following this crack measuring process, crack growth per cycle (da/dN) and change in stress intensity at the crack tip (ΔK) were determined for the stable crack growth regime. These are used in the Paris equation to model crack growth via the defect-tolerant approach (ASTM E647):

(Equation 1) $$\text{da/dN}=\text{C}{\left(\Delta \text{K}\right)}^{\text{m}}$$

The standard equation used for ΔK can be found in ASTM E647-24, Equation (A1.3). These values were used to construct plots of da/dN vs. ΔK. All five samples were used to create a single data set for each material treatment.

To provide a measure of fatigue crack propagation resistance (CPR) of the materials, the right-left shift of the respective da/dN curves in the Paris regime had to be measured. To do this, the log(ΔK) value at 10^−5^ mm/cycle (i.e. log(da/dN) = −5) was found after the data were log transformed and fitted with a linear least-squares regression line. This value corresponded to the 10^−5^ mm/cycle intercept described by Atwood (Atwood et al., 2011) and is related to ΔK_inception_, the stress intensity required for crack growth inception, used as a measure of right-left shift in other studies (Baker et al., 2000, 2003; Gencur et al., 2006). The value of C from the Paris equation (Equation (1)) can be used to provide a similar measure of the shift of the fatigue data by reporting da/dN at ΔK = log(0) = 1. This has been done in studies where all slopes are similar and the data for all material treatments passes through ΔK = 1 (Atwood et al., 2011). In this work, slopes were similar, but ΔK values were consistently above 1, so using the Paris C value as an intercept would amount to an extrapolation, motivating use of the da/dN = 10^−5^ mm/cycle intercept instead. This measure will also be referred to as the “10^−5^ intercept” for the sake of simplicity, and is given by:

(Equation 2) $$I=10^{x}=10^{(-5-a)/b}$$

which is simply the back-transformation of the linear regression of the transformed data in the form *y* = *bx*+ *a*, where a is the y-intercept of the fit of the log-transformed values, b is slope, and y = −5. Similarly, log (ΔK) at 10^−4^ mm/cycle was reported as an additional measure of fatigue crack propagation resistance at a higher crack growth rate. The benefit of this measure over ΔK_inception_ is that it is compatible with the Atwood et al. method of increasing load at regular intervals (Atwood et al., 2011) and is easily adaptable to report the right-left shift of the ΔK vs. da/dN curves across a range of crack growth rates, as will be done in this study for 10^−5^ and 10^−4^ mm/cycle measures.

Comparisons between curves were made in the log-transformed regime using 95% confidence intervals (CIs) constructed in GraphPad Prism. Any overlap in CIs at the transformed crack growth rate of interest (e.g. log(da/dN) = −5) was thus indicative of statistical nonsignificance at the α = 0.05 level while lack of overlap indicated significance. More details on this procedure can be found in the supplemental materials. With five samples for each materials treatment, and an average of about 10 data points per sample, each material treatment had about 50 observations (range: 45 to 55). Since each of these is an observation from the same material treatment, regardless of sample, analysis was performed on all the data points from each material treatment, combined.

### 2.5. Imaging

Optical imaging of fatigue fracture surfaces was completed on a Keyence VHX microscope. Oblique illumination highlighted sample surface topography, while polarized lighting provided color contrast of birefringent areas on the fracture surface. Several polarized images were analyzed in Image J (ImageJ version 1.54g, NIH, USA) by converting to 8-bit grayscale and using an intensity threshold of 120 to separate bright regions from dark. Scanning electron microscopy (SEM) was performed on two different machines. For granule-scale imaging (~400× magnification), a Tescan Vega3 microscope (Tescan Group, Czech Republic) was used with uncoated samples, an accelerating voltage of 1.7 kV, and a working distance of 9–10 mm. For imaging of carbon black-rich regions between polymer granules, a Thermo Fisher Helios 5CX DualBeam microscope (Thermo Fisher Scientific, Waltham, MA) was used after samples were sputter coated with 6 nm of gold/palladium coating. Accelerating voltage ranged from 2 kV–5 kV and working distance was 4 mm.

## 3. Results

### 3.1. Tensile toughness

**Fig. 3.**
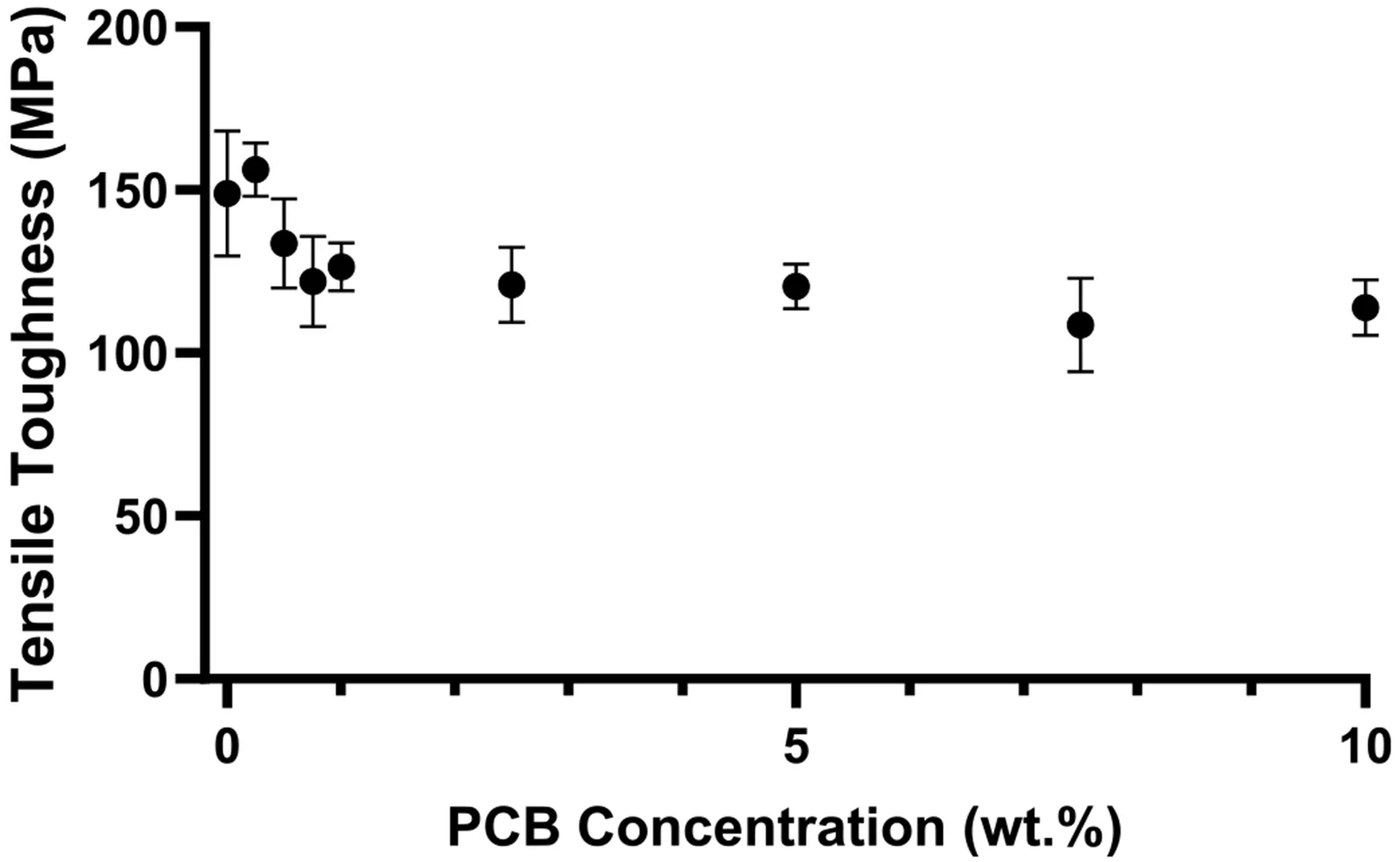
Tensile toughness results for the composites, which all used pigment carbon black (PCB) as the filler material in GUR 1020 UHMWPE.

Tensile toughness results are shown in Fig. 3 where tensile toughness tended to decrease with the addition of carbon black. Brown-Forsythe and Welch’s ANOVA tests indicated significant differences between means, so multiple comparisons tests were performed as described in the methods section. Notable results were as follows: first, the neat tensile toughness result was consistent with that observed for virgin GUR 1020 UHMWPE by Huot et al., who used samples of equivalent geometry. A slight increase occurred from neat to 0.25 wt% PCB, but was not statistically significant (Dunnett’s adjusted p = 0.994). A decrease from 0.25 to 0.5 wt% occurred; this difference was statistically significant (adjusted p = 0.0005). No significant decrease was observed from 0.75 wt% to 10 wt% in the multiple comparisons test (adjusted p = 0.84). To further investigate, a focused Welch’s *t*-test was performed on the 0.75 wt% and the 10 wt% samples, confirming lack of statistical significance, albeit barely (p = 0.068). A summary of all multiple comparisons results for tensile toughness can be found in the supplemental materials (S1).

**Fig. 4.**
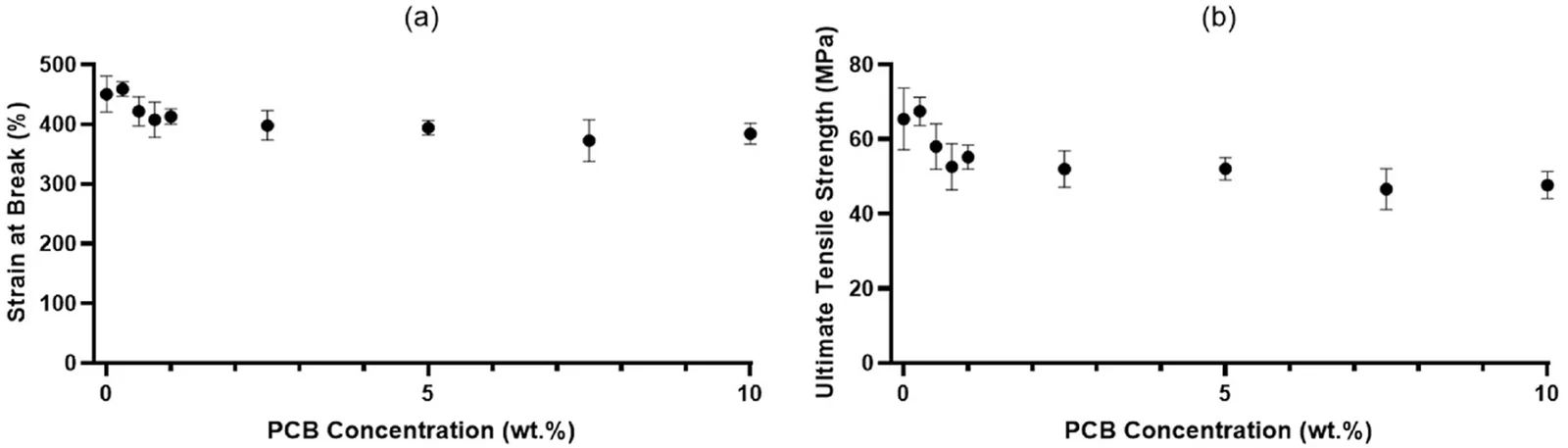
Other tensile results: (a) engineering strain at break (i.e., elongation at break), (b) ultimate tensile strength.

Fig. 4 shows several other results obtained during tensile testing. Strain at break and ultimate tensile stress followed similar trends to tensile toughness, consistent with the fact that these two measures directly influence area under the stress-strain curve (tensile toughness). Both measures exhibit limited decreases even with large increases in filler content, with a 6% decrease for strain at break and a 9% decrease for UTS from 0.75 wt% to 10 wt% carbon black content. The small reduction in ductility across a range of increasing filler loadings is consistent with recent observations in the literature utilizing other additives to UHMWPE (Inverardi et al., 2025).

### 3.2. Impact toughness

**Fig. 5.**
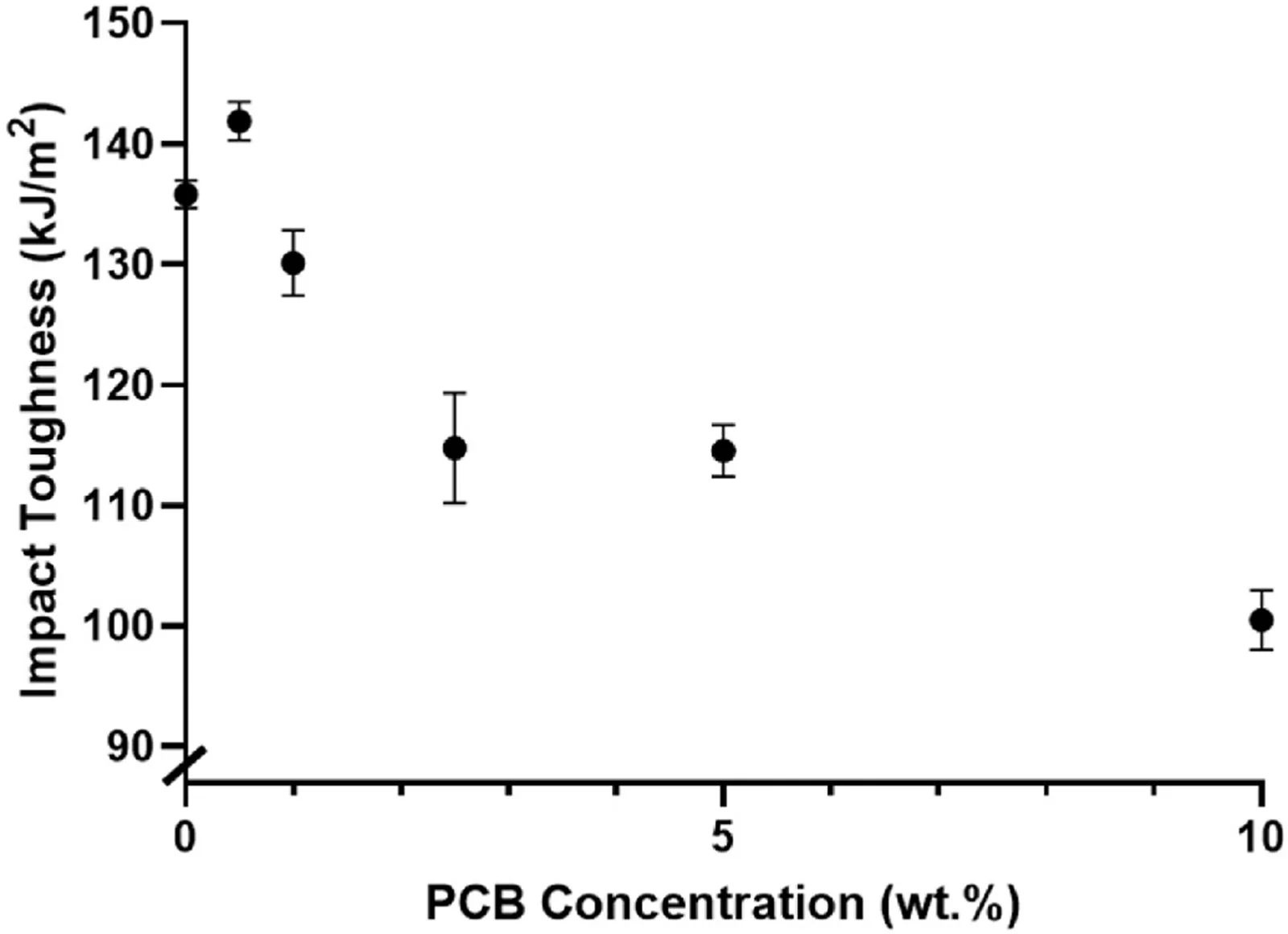
Impact toughness results. Relevant statistical comparisons are highlighted in the text. Note that several intermediate concentrations added for tensile testing do not exist for the impact results.

Impact toughness results (Fig. 5) demonstrate similar trends to tensile toughness. With small errors associated with these tests, all pairs of material treatments exhibited statistically-significant behavior in the multiple-comparisons test except for Neat vs. 1.0 wt% (adjusted p = 0.07) and 2.5 wt% vs. 5.0 wt% (adjusted p > 0.99). As a note, the behavior of the neat material here is similar to the 134 kJ/m^2^ observed in a previous study for virgin GUR 1020 UHMWPE (Huot et al., 2011).

### 3.3. Fatigue crack propagation resistance

**Fig. 6.**
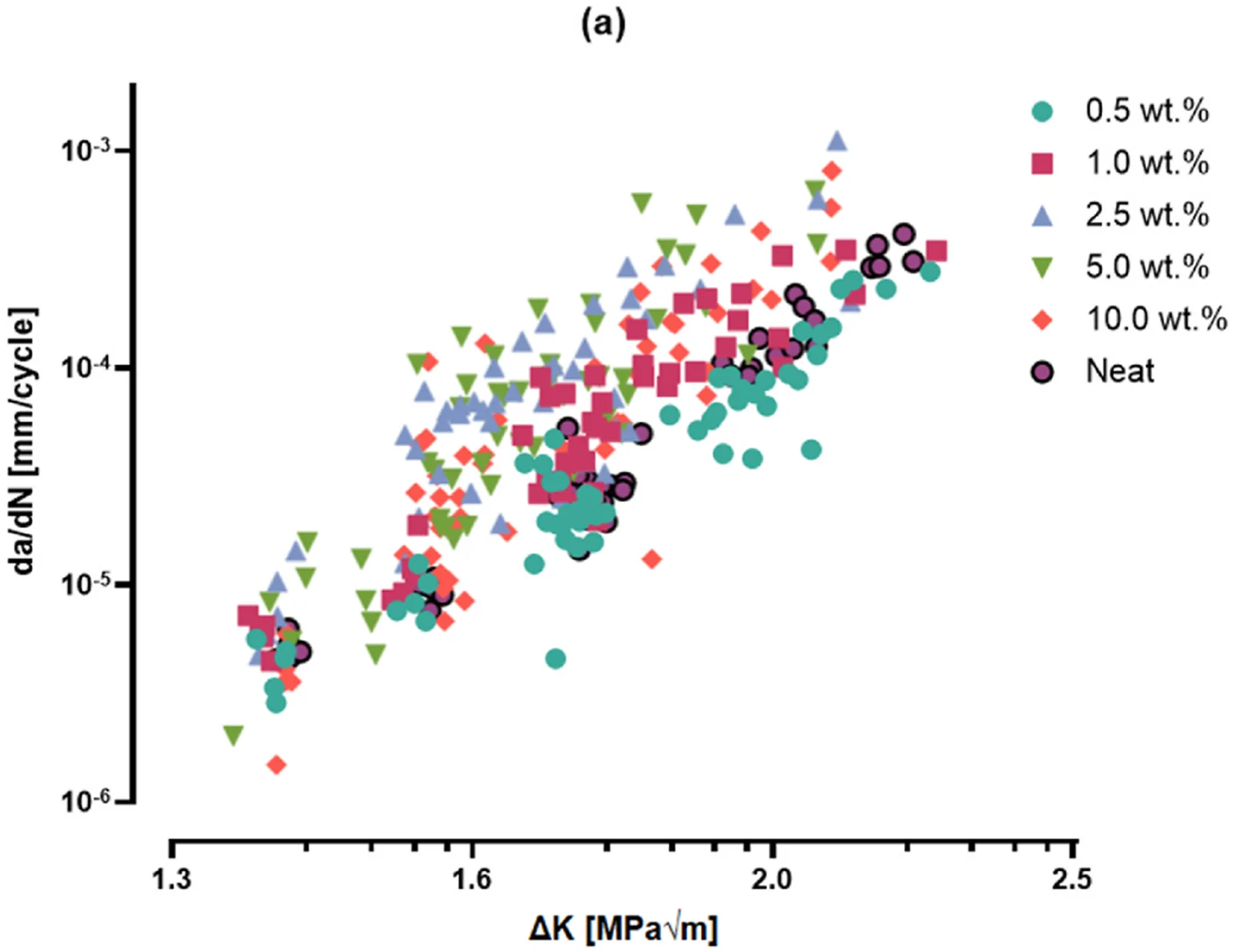
Results from defect-tolerant fatigue testing: crack growth per cycle (da/dN) vs. change in stress intensity at the crack tip (ΔK).

**Fig. 7.**
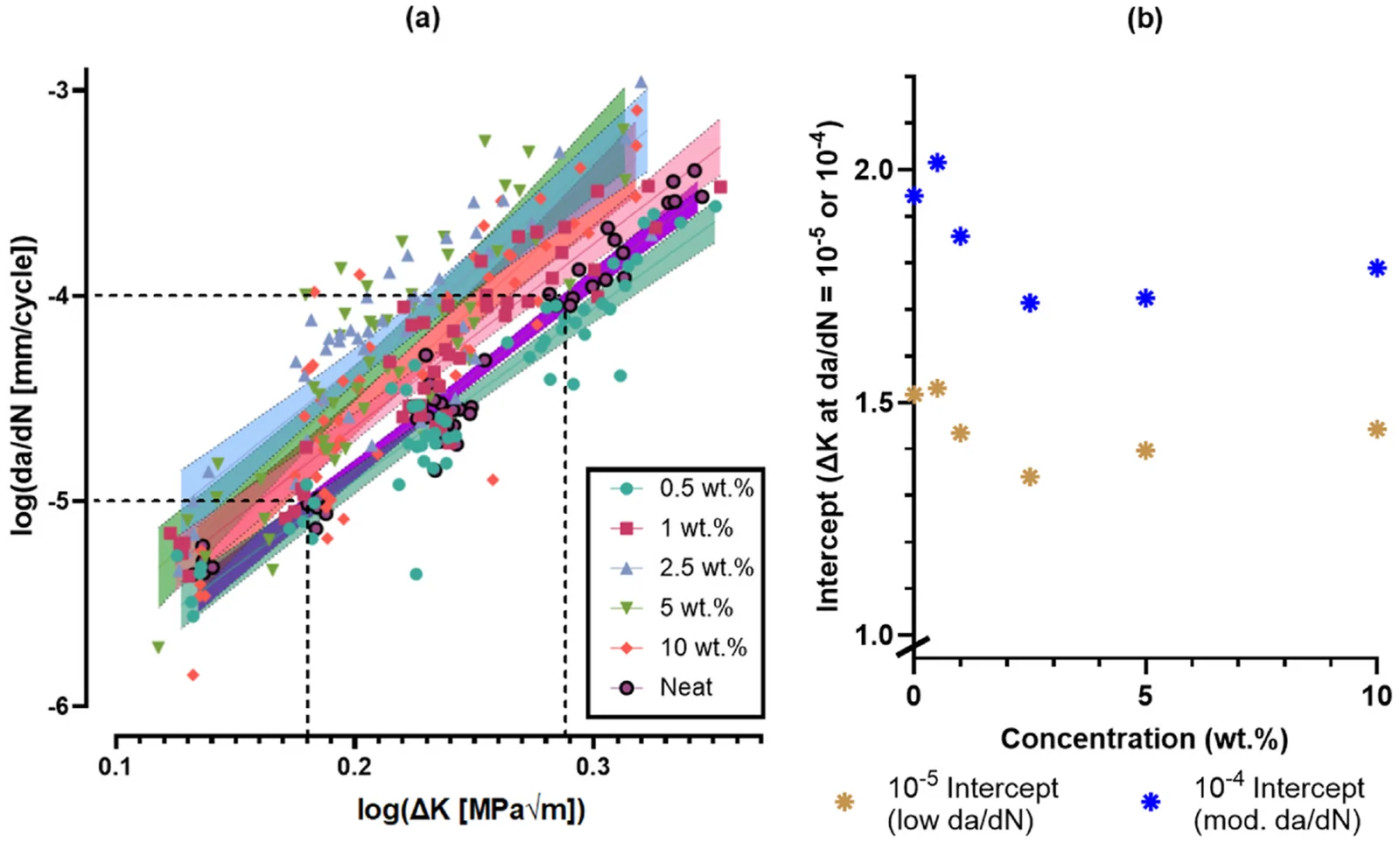
(a) log_10_-log_10_ transformed fatigue results with 95% CIs indicated by shaded regions, plus dotted lines showing how the 10^−5^ and 10^−4^ intercepts were found for the neat material in purple. (b) Fatigue results as indicated by stress intensity factor ΔK [MPa√m] necessary to yield a crack growth rate of 10^−5^ or 10^−4^ mm/cycle. Note that these are back-transformed from the values on the x axis in (a), e.g. 10^0.18^ ≈ 1.5 for the neat 10^−5^ intercept. (For interpretation of the references to color in this figure legend, the reader is referred to the Web version of this article.)

Fatigue crack propagation da/dN plots for the composite materials and the neat control are shown in Fig. 6. As described in the methods section, the right-left shift of this Paris regime data is used to determine the resistance to fatigue crack propagation of these materials, with curves farther to the right exhibiting better resistance to fatigue crack propagation, assuming similar slopes in the bilogarithmic-transformed data. The fitted slopes of the material treatments were similar to each other in this study (range: 8.3 to 10.9), though an analysis of covariance revealed that there were some slight statistical differences in slopes (p = 0.042). Slopes are listed in Table 2 with their associated standard error values, as computed in GraphPad Prism. The linear regression lines representing these slopes are displayed in Fig. 7a, along with shaded 95% confidence intervals of those predictor lines.

Fig. 7b was produced as described in the methods and shows both the 10^−5^ and the 10^−4^ intercept values, representing the materials’ resistance to fatigue crack propagation at two different crack growth rates. Error associated with these values can be understood based on the 95% CIs for the linear regression predictor lines, indicated by the shaded regions around the best-fit lines in Fig. 7a. Further description and discussion of error can be found in the supplemental materials.

The shapes of the plots in Fig. 7b indicate an uptick in fatigue crack propagation resistance with addition of a small amount of carbon, followed by expected decreases with the addition of carbon black. However, the higher concentrations deviate from this behavior, with the shape of the plot indicating an increase in fatigue crack propagation resistance from 2.5 wt% to 10 wt% pigment carbon black. This behavior was observed at both crack growth rates. Results of 95% CI comparisons to determine statistical significance of the pairings at the two crack growth levels are included in the supplemental materials.

### 3.4. Combined toughness results

**Fig. 8.**
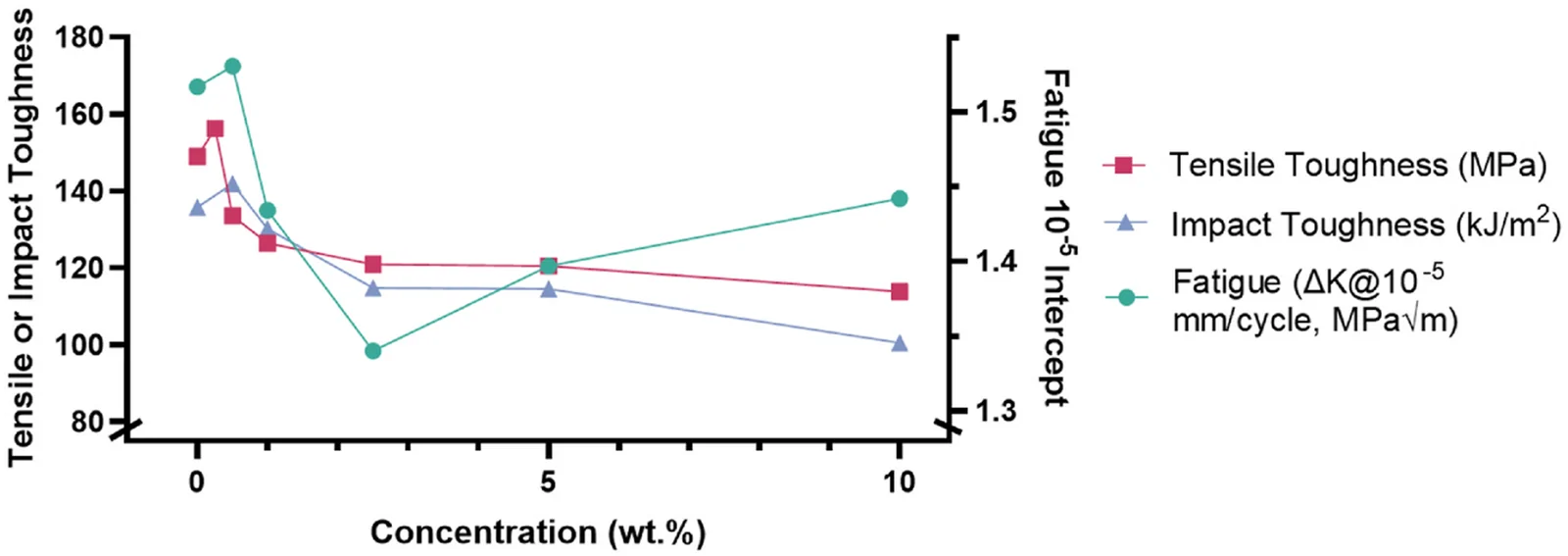
Combined toughness measures with respect to concentration, including the six material treatments for which all three toughness measures were obtained, with the 0.25 wt% treatment added for tensile toughness as well. Indications of error for these three measures have been included in other figures and are left out here.

Combined toughness results are shown in Fig. 8 for neat, 0.5 wt%, 1 wt%, 2.5 wt%, 5 wt%, and 10 wt% filler loading levels. The 0.25 wt% tensile result is included as well, though no impact or fatigue samples were collected at that concentration. For visual clarity, error bars are left off here but were included or discussed in previous figures. These results demonstrate similarity in material behavior across the three measures of toughness at low additive concentration, where an uptick in toughness is observed followed by a sharp decrease. At higher concentrations, the similarity of behavior between tensile and impact toughness is evident, as is the divergence of fatigue results.

**Fig. 9.**
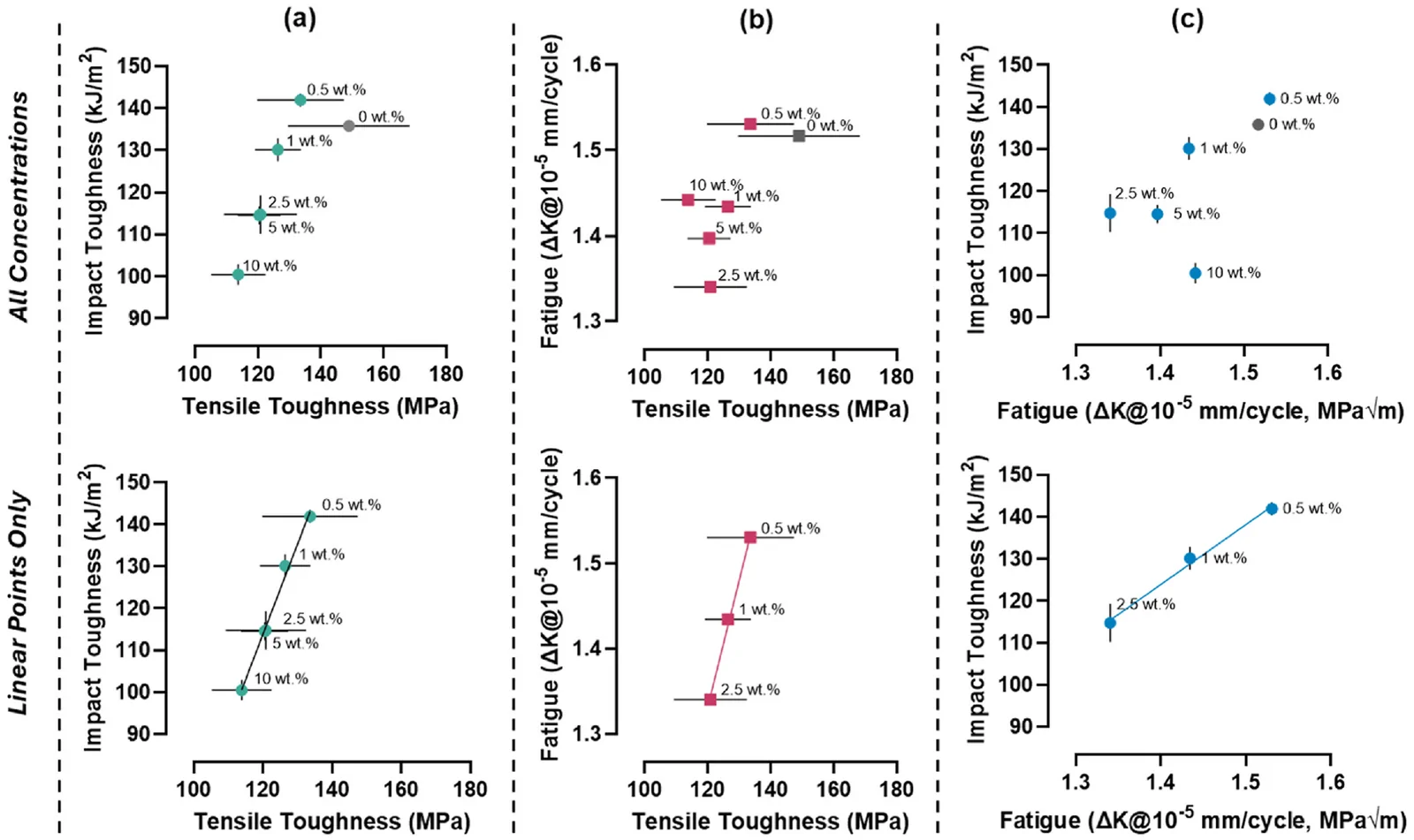
(a,b,c) Pairwise comparisons of toughness measures, with the linear portions repeated in the lower plots. Note that the 2.5 wt% and 5.0 wt% results were nearly identical for both tensile and impact toughness. The gray marks represent the neat control, which had a very small error bar for impact toughness. Due to the logarithmic nature of the fatigue results, no error bars can be assigned but a discussion of error is included in the supplemental materials.

Pairwise comparisons of the three toughness measures are shown in Fig. 9. Tensile and impact toughness were linearly correlated among the five composite material treatments available for comparison (R^2^ = 0.96), as shown in Fig. 9a. The behavior of the neat control material tended to deviate from that of the composites. Other comparisons of toughness measures are included in Fig. 9b and c, demonstrating linear relationships only from 0.5 wt% to 2.5 wt% PCB additive. Observation of fracture surfaces and material microstructure, covered in the next sections, can provide further information about the mesoscale material behavior that led to the measurements presented here.

### 3.5. Fracture surface imaging

Fracture surface images can visually demonstrate the fracture surface topology and composition in these composites. Images are provided for both impact and fatigue samples in this section and provide a qualitative indication of the crack propagation behavior of the materials under these testing modalities.

**Fig. 10.**
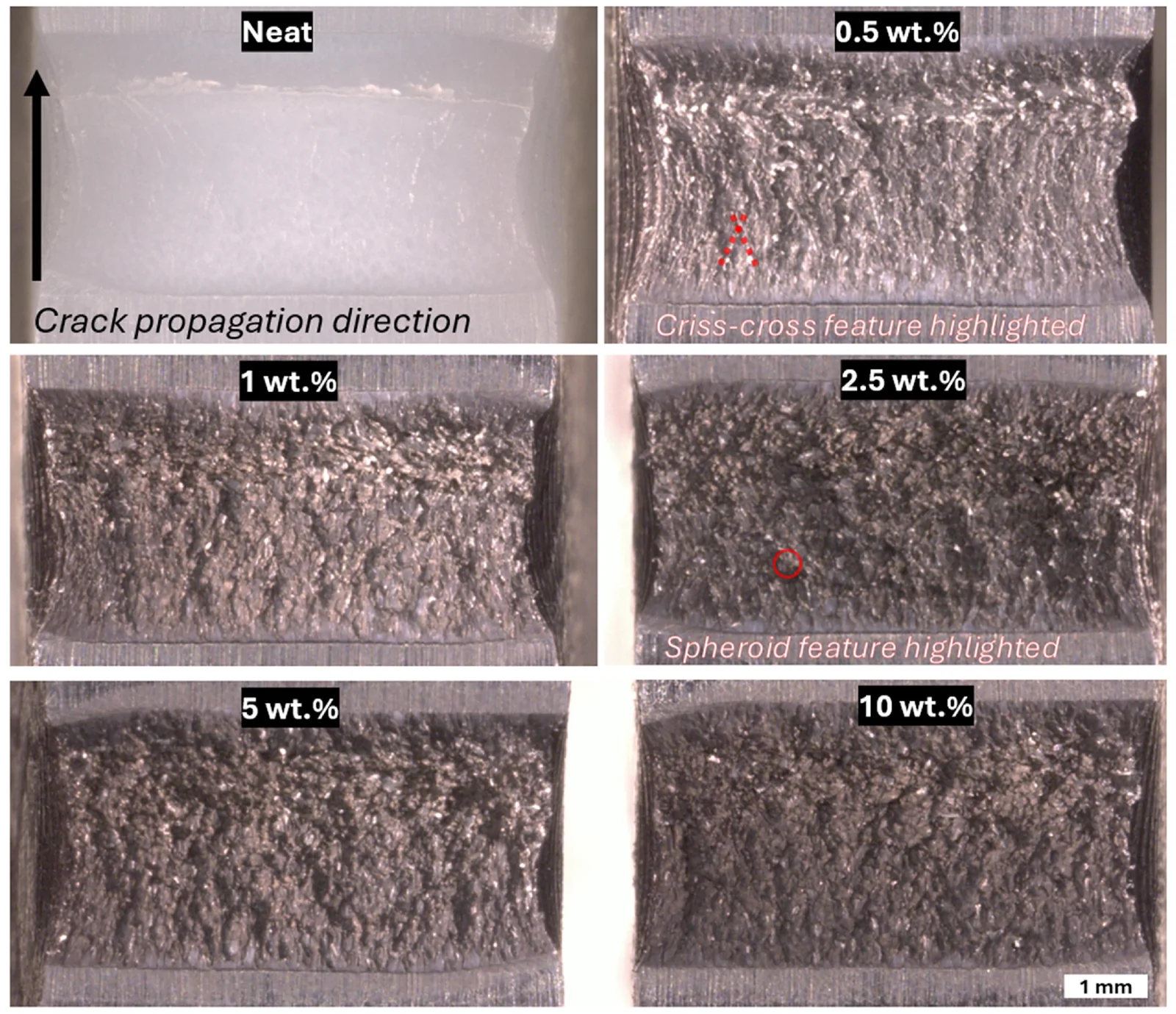
Representative impact fracture surfaces via optical microscopy using oblique illumination. Examples of criss-cross and spheroid features are highlighted. Note that the neat sample contains criss-cross features characteristic of ductile tearing, though these features are difficult to see in this image.

Representative impact fracture surfaces are shown in Fig. 10 and exhibit a clear difference in topography between neat and 0.5 wt% PCB, followed by what appear to be incremental changes with increases in carbon black content after that. The neat sample exhibits the “criss-cross” tearing marks characteristic of ductile transgranular fracture in UHMWPE (Ansari et al., 2016). “Transgranular” will be used here to describe fracture through the polymer granules, as opposed to “intergranular” fracture through the filler-rich granule boundary regions. These criss-cross features become highly prominent with greater surface roughness on the 0.5 wt% and 1 wt% samples before becoming less prominent as PCB concentration is increased to higher levels. At the higher concentrations, there appears to be a transition away from these transgranular ductile tearing features. Instead, intergranular fracture occurs, as evidenced by the presence of more spheroid-type features matching the scale of polymer granules (100–200 μm diameter). One example of each feature type is highlighted in red in Fig. 10.

**Fig. 11.**
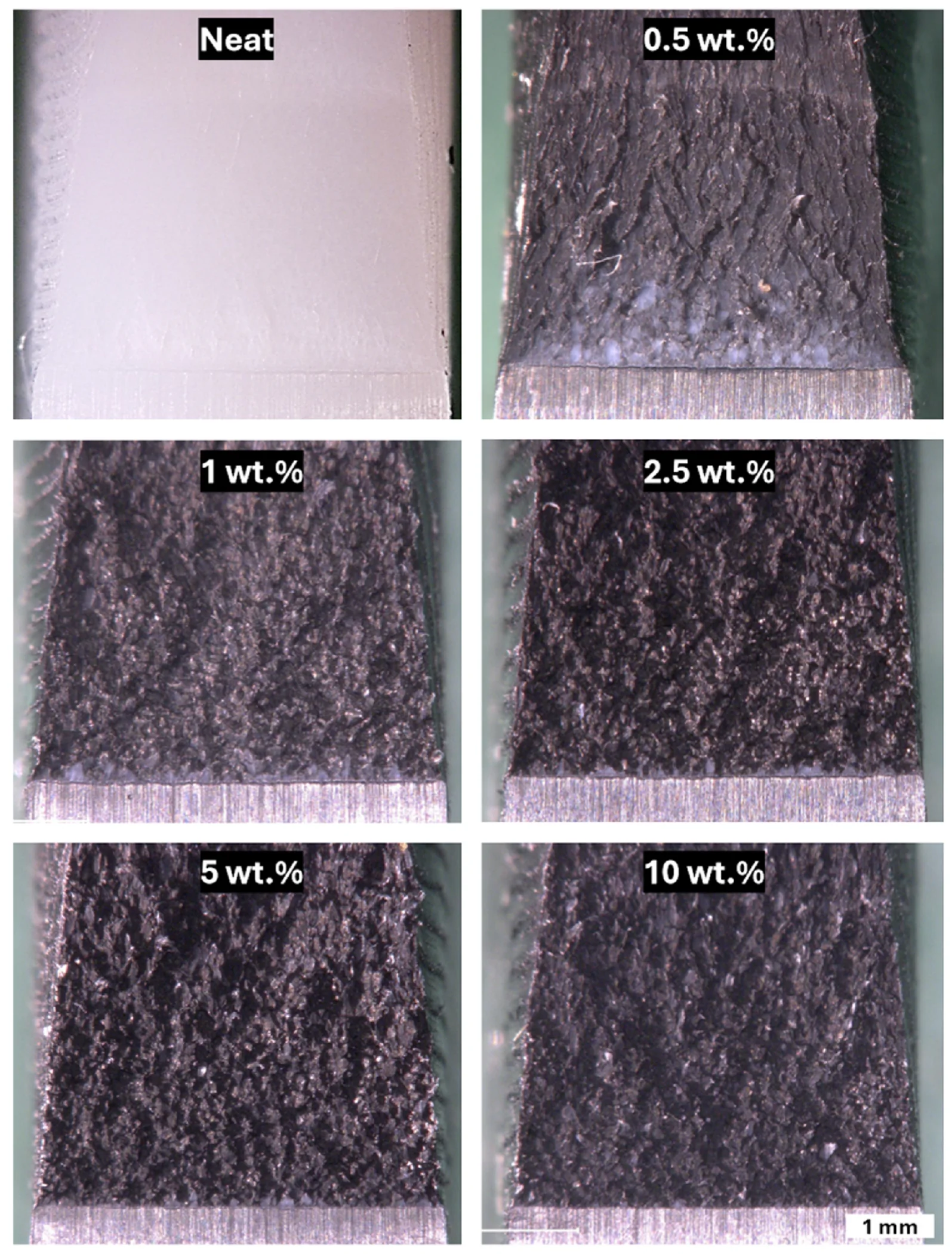
Fatigue fracture surface images, highlighting topographical differences among representative samples at each concentration. The razor cut is visible as a smooth region at the bottom of each image, with the crack beginning just above that region.

Representative fatigue fracture surface optical images emphasizing the topographical features of the surface are shown in Fig. 11. As with the impact samples, criss-cross marks are present on the surface of the neat material here. The 0.5 wt% material again exhibits an increase in prominence of these criss-cross features and other evidence of multi-granule ductile tearing, while also exhibiting a rougher surface than the neat material. Above 0.5 wt%, a distinct shift toward intergranular fracture occurs, with no obvious large multi-granule tearing features for concentrations above 0.5 wt%. At 1 wt% and above, there appear to be minimal differences in fracture surface topographical features in these images.

**Fig. 12.**
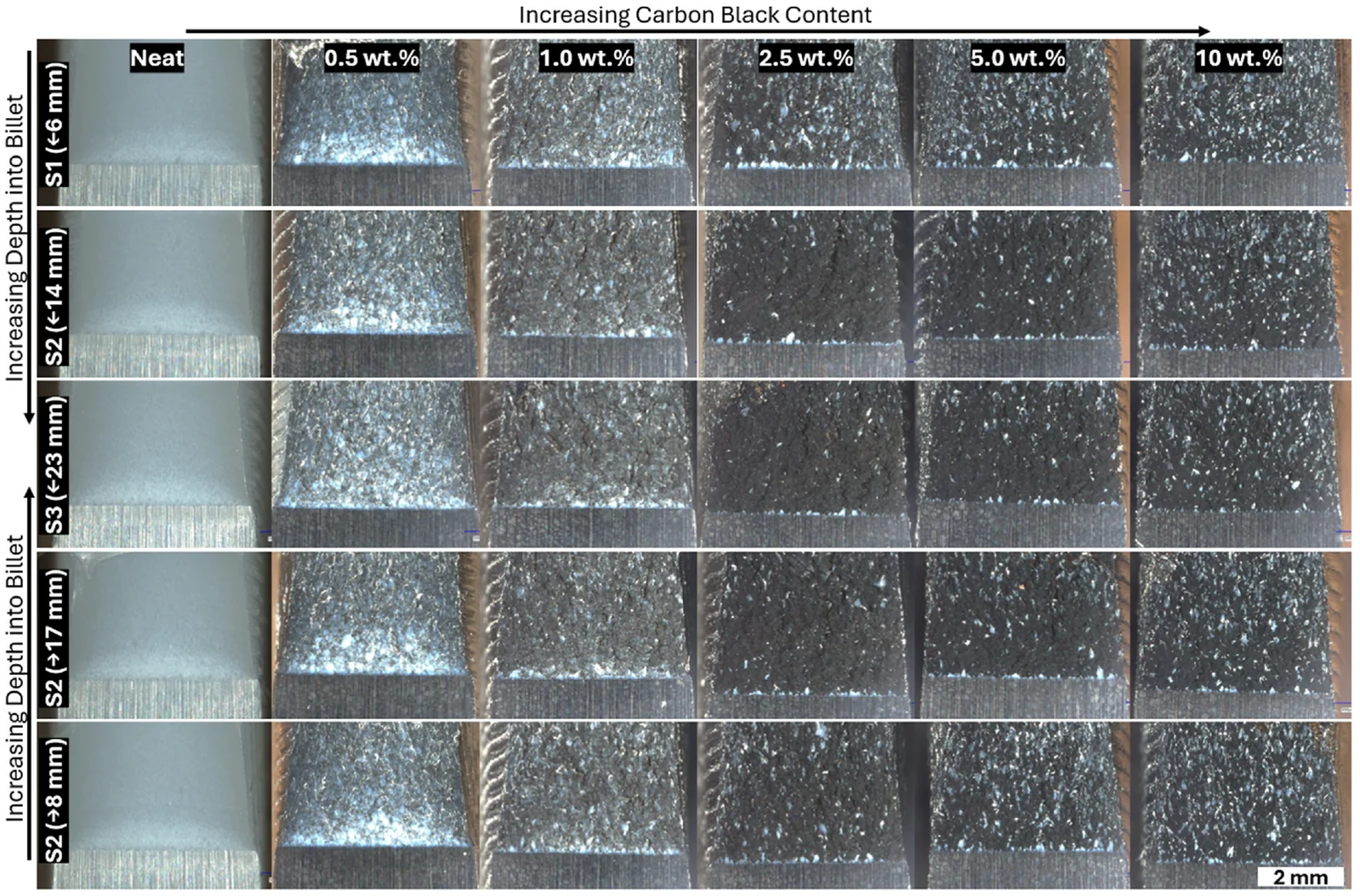
Polarized optical images of all fatigue fracture surfaces. Approximate nearest distance from each billet’s outer edge to the center of each sample is provided in the labels on the left.

**Fig. 13.**
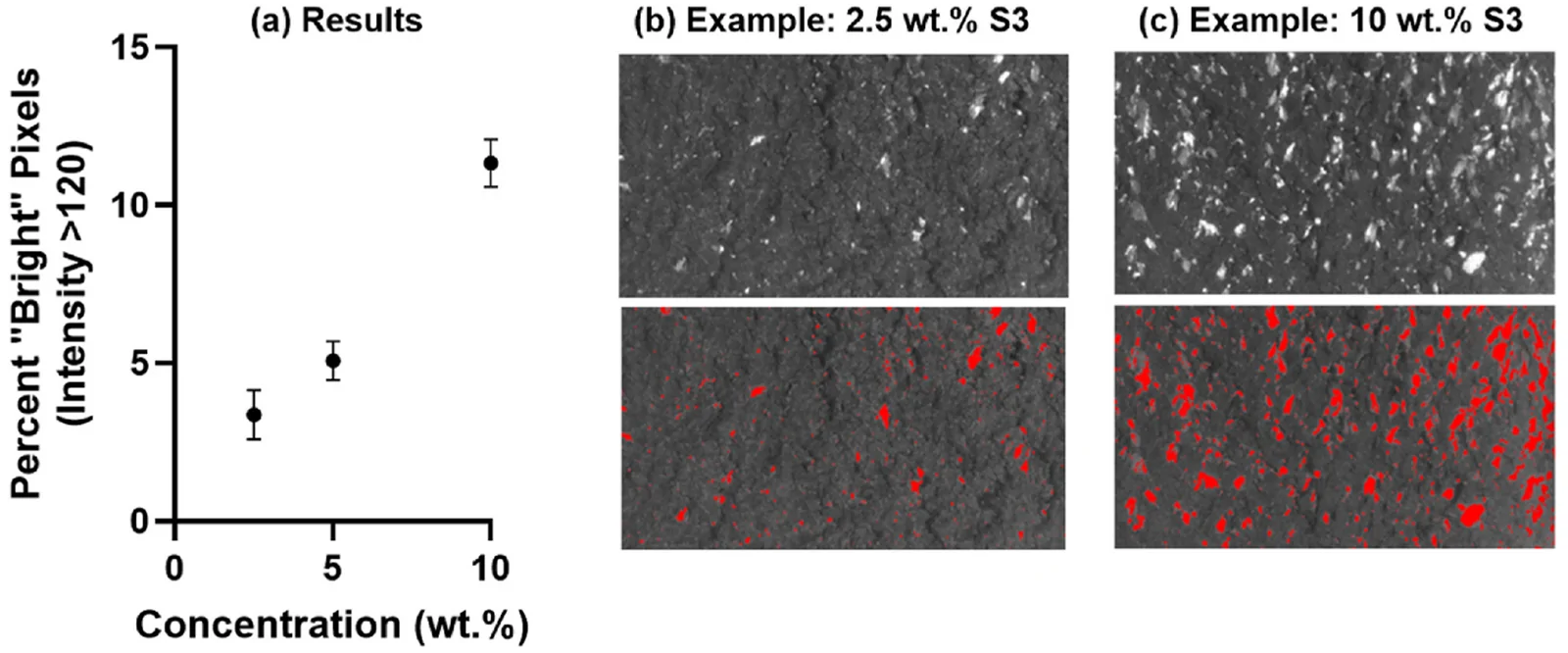
Image analysis of bright regions occurring in higher-concentration materials’ fracture surfaces (n = 3; S2, S3, and S4 used here for each concentration of interest to show distinct behavior for samples taken from within the depth of the billet). (a) Quantified results. (b) Representative sample for 2.5 wt% showing regions with pixel grayscale intensity >120 in red. (c) Representative sample for 10 wt%. (For interpretation of the references to color in this figure legend, the reader is referred to the Web version of this article.)

Polarized optical fatigue fracture surface images are provided in Fig. 12 for all five samples at each concentration tested. These images draw attention to the composition of the visible surface on these samples. Several transitions are notable, including a darkening of the fracture surfaces from 0.5 wt% to 2.5 wt%, consistent with higher levels of exposed carbon black from the filler-rich intergranular regions. From 2.5 wt% to 10 wt%, while the surface background remains dark, isolated bright regions become more frequent with the increases in concentration. This is especially the case for samples from the center of the billet, i. e., S3 (see Fig. 13). These bright regions represent localized ductile tearing of polymer granules (transgranular crack propagation, but through individual granules instead of multiple granules as observed at the lowest concentrations).

### 3.6. SEM imaging

**Fig. 14.**
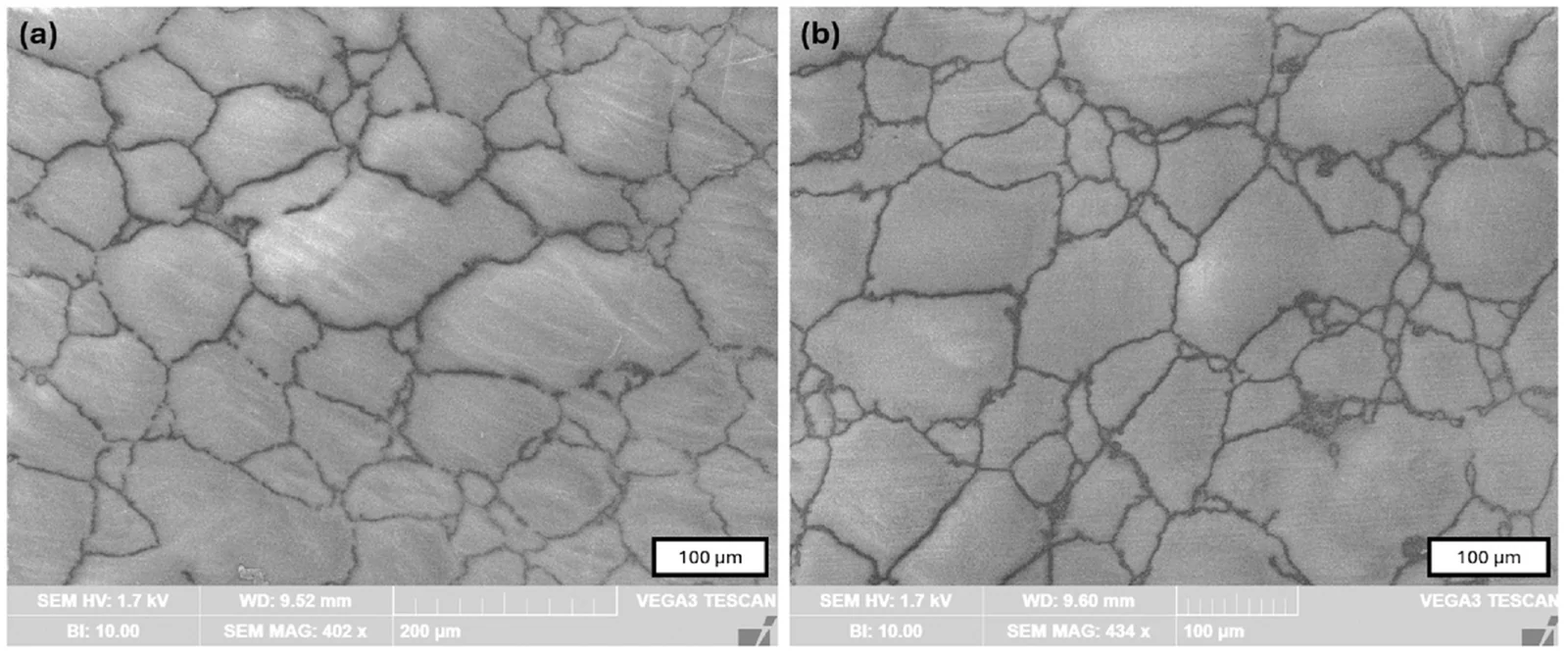
Onset of complete granule coating observed between (a) 1 wt% and (b) 2.5 wt% compositions in uncoated scanning electron microscopy (SEM) images.

Scanning Electron Microscope (SEM) images were taken of thin sections and observed serially to understand the nature of the granule boundaries in the materials. Fig. 14 shows SEM images of unaltered 1 wt % and 2.5 wt% sections. These show that polymer granules were only partially coated in carbon black at 1 wt%, as indicated by frequent gaps in the black granule boundaries in Fig. 14a. On the other hand, complete or near-complete granule coating was achieved by 2.5 wt% (shown in Fig. 14b) and all concentrations above 2.5 wt%, with very few gaps present in the black filler-rich granule boundaries.

**Fig. 15.**
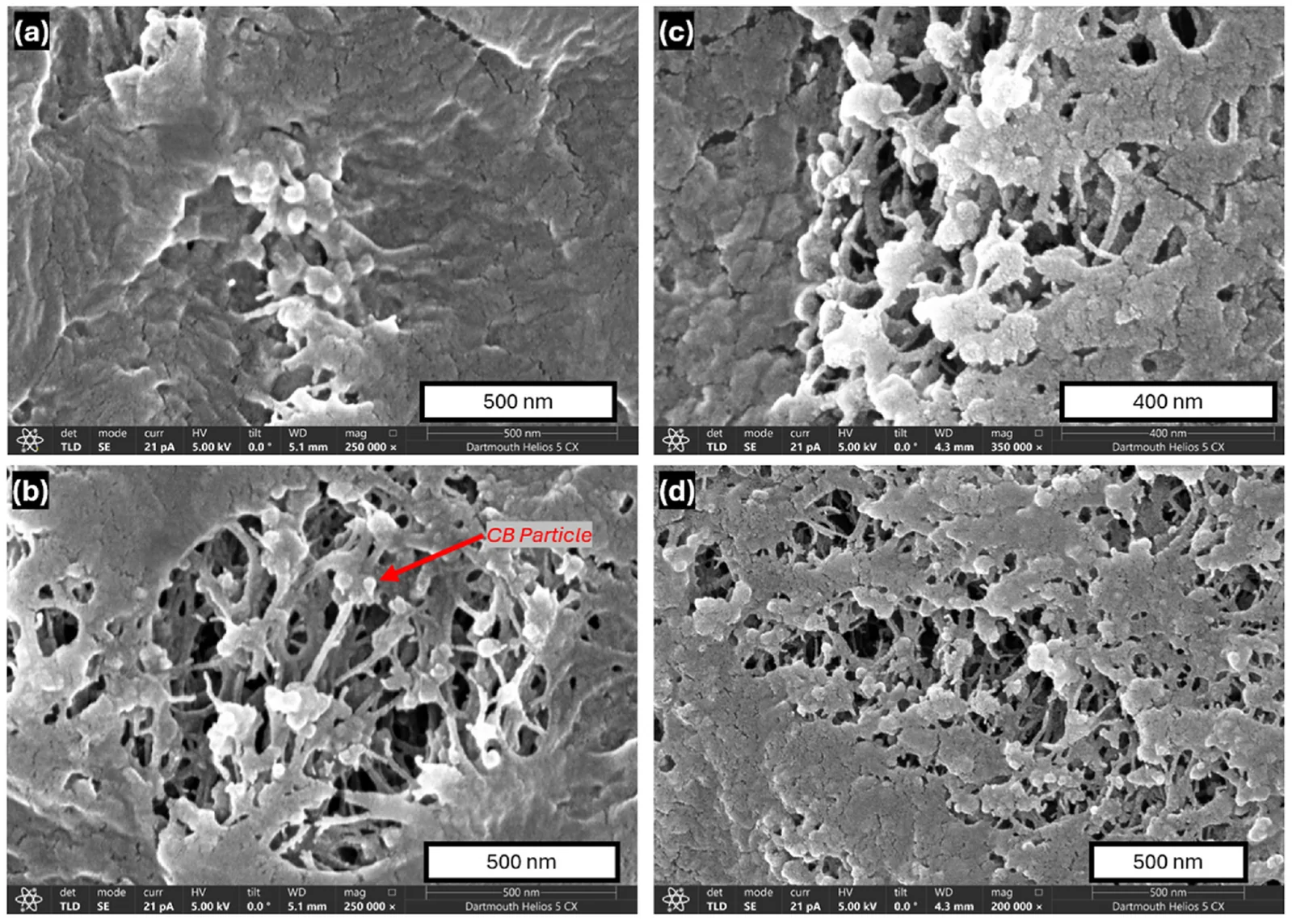
High magnification SEM images of 1 wt% (a,b) and 10 wt% (c,d) thin sections held at 100% elongation (direction of strain: vertical). One of the many visible carbon black nanoparticle spheres is indicated with an arrow.

**Fig. 16.**
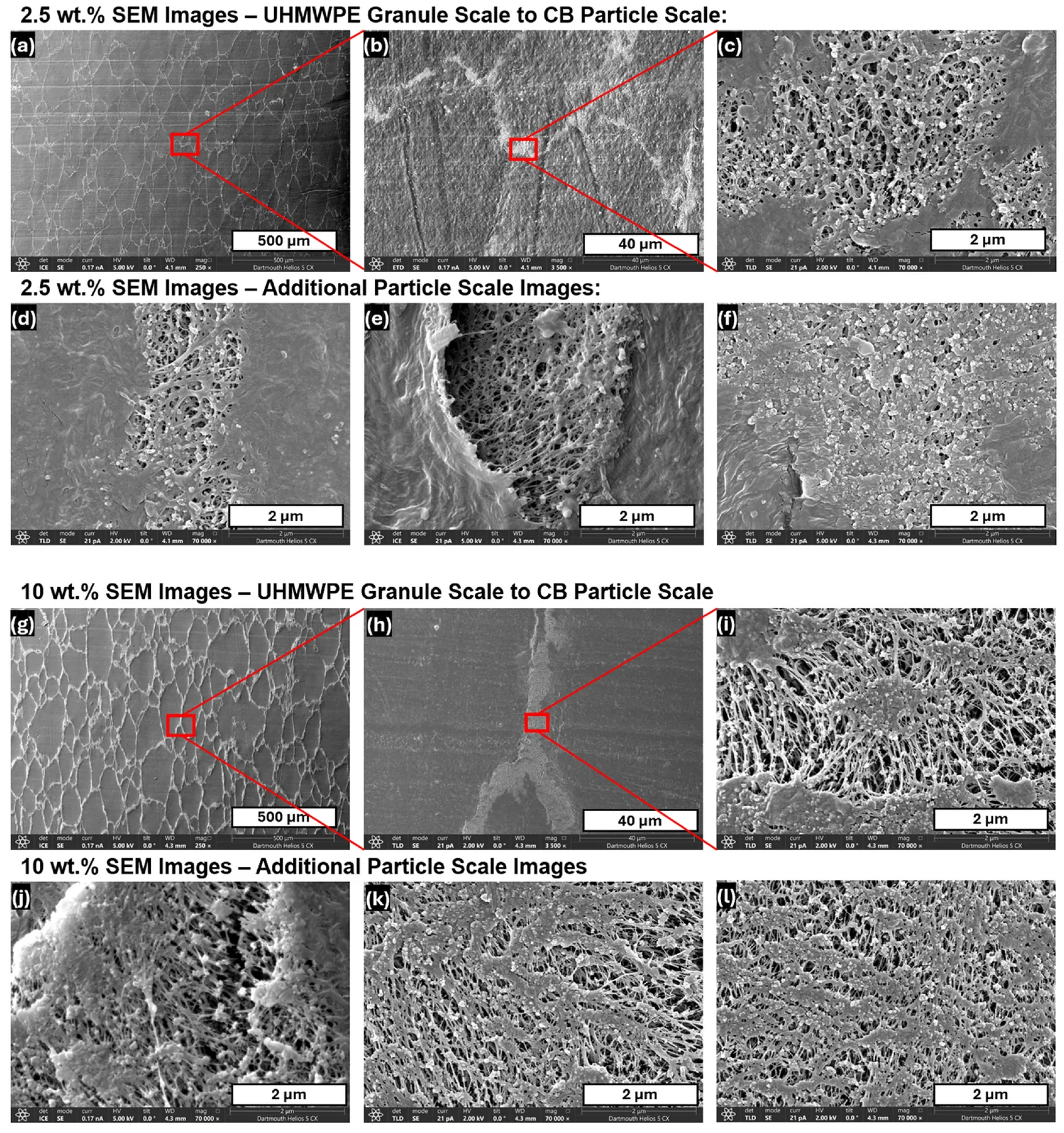
SEM images of 2.5 wt% compared to 10 wt% materials held at 100% elongation (direction of strain: vertical).

Fig. 15 shows 1 wt% and 10 wt% materials, which were strained to 100% engineering strain, clamped in place at that strain, and imaged following sputter coating. These images exhibit a clear feature of these composites: the polymer interacts very highly with the carbon black in the intergranular regions – a behavior that becomes especially clear in images of strained materials like these. This is the case at both lower concentrations such as 1 wt% and higher concentrations such as 10 wt%. Furthermore, these granule regions exhibit some void-like behavior; for example, the image in Fig. 15b is like a small spherical void with polymer fibrils transecting the open region. At concentrations of 0.5 wt % and 0.25 wt% (not shown), void-like granule boundary features like this were not observed readily under SEM, instead becoming apparent starting at a concentration of 1 wt%. Such features become even more prominent at higher concentrations, such as 10 wt% (Fig. 15c and d). Comparisons between another pair of strained samples, 2.5 wt% and 10 wt%, are shown in Fig. 16 showing subtle differences in fibril characteristics at the different concentrations.

## 4. Discussion

Historically, tensile, impact, and fatigue toughness have been linearly correlated in UHMWPE and its crosslinked variants, as microstructural changes in those materials led to decreases in measures of toughness with increasing crosslink density (Baker et al., 2003; Gencur et al., 2006; Huot et al., 2011; Morrison and Jani, 2009). While the mechanical behavior of crosslinked materials is well understood, the behavior of high viscosity, phase segregated UHMWPE polymer nanocomposites is not as well understood. With increased interest in solid-state composites of UHMWPE, this study characterized the mechanical behavior of these materials across three measures of toughness. The toughness values attained in this study demonstrate promise for using solid-state nanocomposites of UHMWPE in high-load applications such as orthopedic bearings, with properties that are at times even superior to the neat materials and vastly higher than some previously-published carbon-UHMWPE composites (Favreau, 2022). However, the greater value of this study’s findings may lie in the nuanced understanding of toughness behaviors these results can begin to provide for high-viscosity polymer nanocomposites, which have broader relevance across similar material categories. While the overarching objective of this work was to better understand mechanical behavior in solid-state composites of UHMWPE via a set of clinically-relevant tests, more specific objectives sought to lay the foundation for continued study of microstructural reasons for those observed behaviors. To that end, they included: (1) to understand how increases in filler concentration affect tensile toughness, impact toughness, and fatigue crack propagation resistance differently, and (2) to identify potential microstructural reasons for observed increases or decreases in toughness measures at (a) high, (b) low, and (c) moderate concentrations. This discussion will address these points in separate sections.

### 4.1. Relationships between toughness measures are not all positive correlations (objective 1)

In the pairwise correlations of toughness measures (Fig. 9), we found that impact toughness, tensile toughness, and fatigue CPR of pigment carbon black-UHMWPE composites were linearly correlated until 2.5 wt % PCB, above which, only impact and tensile toughness remained linearly correlated. Published work in the literature has demonstrated that tensile and impact toughness are positively and linearly correlated in crosslinked forms of UHMWPE (Huot et al., 2011). Likewise, impact toughness and fatigue CPR are known to be positively correlated across various levels of crosslinking (Doshi et al., 2016). Such behavior has been observed in a recent set of studies of a drug-loaded UHMWPE composite, wherein the presence of the additives led to decreases across impact toughness, fatigue CPR (albeit with only one composite material treatment reported), elongation at break, and UTS, the last two of which will largely determine tensile toughness (Asik et al., 2025; Inverardi et al., 2025). However, here the relationship between fatigue and the other two measures deviated from these positive correlations starting at 2.5 wt%, coinciding with the onset of complete granule coating in these composites. The onset of complete granule coating has been shown to have implications for macroscopic property changes in other studies of UHMWPE composites (Puertolas et al., 2022; Senatov et al., 2014), and has been shown to occur at a similar level of filler loading to that observed in this study (Puertolas et al., 2022). While the present study does not seek to prove causation regarding this effect, in the next sections we elaborate on microstructural factors that may have led to the relative increases in fatigue CPR at the two highest concentrations tested. These microstructural factors may help to elevate the materials’ fatigue CPR, relative to tensile and impact toughness, due to the importance of stress distribution and deformation characteristics at the cyclically loaded crack front.

### 4.2. Fatigue behaviors at high concentrations: 10 wt% higher than 2.5 wt %. (Objective 2a)

#### 4.2.1. Granule-scale plastic deformation appears greater at 10 wt% than 2.5 wt%

At high concentrations, fatigue crack propagation resistance was maintained or even increased compared to some moderate concentrations, unlike tensile and impact toughness. The increase from 2.5 wt% to 10 wt% was statistically significant as determined via the method described in the supplemental materials. Representative images highlighting the surface topography of the fatigue samples (Fig. 11) demonstrated no notable differences between the 2.5 wt% and the 10 wt % samples. However, in the polarized fracture surface images (Fig. 12), the 10 wt% materials generally show many more exposed, torn polymer granules than those in the 2.5 wt% samples. This suggests an increased propensity for granule-scale plastic deformation in the composites at the highest concentrations. When local regions of plastic deformation occur within filled polymer composites, increases in toughness are known to occur. For example, Fu et al. reported on particulate-polymer composites and suggested that improvements in toughness can be attained when regions of plane stress are provoked between polymer particles, causing local yielding of the material and manifesting as localized plastic deformation or crazing (Fu et al., 2008). Thus, here, the increase in full-granule plastic deformation at 10 wt% over 2.5 wt% may be contributing to the increases in fatigue CPR of that higher-concentration material. These differences in extent of granule tearing were exaggerated when comparing the samples from the center of the billet (e.g., S3, Fig. 12), which may be further contributing to the magnitude of the observed effect.

#### 4.2.2. Potential for crack tip bridging different at 10 wt% than 2.5 wt%

SEM imaging shown in Fig. 16 demonstrates that in both the 2.5 wt% and 10 wt% PCB billet, intergranular regions are thoroughly transected with polymer fibrils. However, in the 2.5 wt% sample, these fibrils appear slightly thicker and less deformed than at 10 wt%, where they appear more plastically deformed, as indicated by a “stringy” appearance with a general alignment towards the direction of applied strain (vertical) in these elongated samples. This suggests that the intergranular domains at 10 wt% are behaving in a more ductile manner than at 2.5 wt% and are likely contributing a significant proportion of the overall plastic deformation in the material.

For monotonic tensile loading (during tensile and impact testing), the plasticity in the intergranular regions for the 10 wt% material may not be giving it an advantage. However, under cyclic loading, a longer crack tip bridging zone enabled by long, ductile polymer fibrils near the crack front can be expected to distribute stress and therefore reduce crack propagation rates compared to a shorter crack tip bridging zone (Ritchie et al., 1989). The mechanism by which this would happen is the following: all else equal, compared to a shorter fibril, a longer fibril can lengthen more (higher ΔL) without reaching ultimate strain, according to simple strain principles: strain = ΔL/L_0_. Given a constrained crack tip opening distance, longer fibrils at the crack tip will not reach their ultimate strain as readily as shorter fibrils. Thus, in the 10 wt% material, with its larger intergranular regions compared to 2.5 wt%, there may be many more unruptured fibrils in the fatigue crack tip vicinity working to distribute stress at the crack tip. Consistent with the results observed here, stress distribution via fibrous crack tip bridging has been observed in carbon nanofiber UHMWPE composites, carbon nanotube-epoxy composites, and many other materials, which show improved fatigue crack propagation resistance as a result (Ghosn, 1994; Lin and Kao, 1995; Liu et al., 2020; Zhang et al., 2007).

In the 2.5 wt% material, a higher effective stress intensity at the crack tip due to a shorter crack tip bridging region may allow crack growth to occur in a more localized manner through granule boundary regions. This could explain the high level of intergranular fracture for 2.5 wt% (darker surfaces, Fig. 12).

Finally, we consider the impact toughness at high concentration: this measure exhibited decreases at high concentration, unlike fatigue CPR, despite having similar characteristics (i.e. an existing Mode I crack being extended via tensile loading). The distinct difference in results, however, might be expected: Zhang et al. observed that the benefit of crack tip bridging decreased as stress intensity at the crack tip grew large (Zhang et al., 2007). With the Izod impact test representing an extremely high stress intensity at the crack tip, given that complete rupture occurs in a single cycle, it is reasonable to expect that impact toughness may not benefit from this stress distribution mechanism as much as the fatigue CPR.

### 4.3. Increases in toughness observed at very low filler concentrations (objective 2b)

At some very low concentration, increases were observed in each of the three measures of toughness. While this increase in toughness was only statistically significant for impact toughness and for the 10^−4^ intercept measure of fatigue CPR (see S3 in supplemental materials), this behavior is still worth discussing as it aligns with some previous observations in the literature (Rama Sreekanth and Kanagaraj, 2013). Fracture surface imaging of impact and fatigue samples showed increases in fracture surface roughness with maintenance of fracture type (transgranular) from neat to 0.5 wt% materials. Thus, with neat and 0.5 wt% fracture surfaces composed entirely or almost entirely of the same material phase (polymer), a higher surface roughness will correspond to a higher-energy surface, consistent with the higher observed material toughness (Griffith, 1921; Pramanik et al., 2012). Without similar observations available for tensile samples, the slight increase in tensile toughness observed from neat to 0.25 wt% materials cannot be elucidated by these results. However, similar behavior has been previously observed in UHMWPE composites, wherein material tensile toughness increases up to some initial filler concentration, then decreases (Rama Sreekanth and Kanagaraj, 2013). The precise origin of this toughening effect cannot be determined with certainty from these results, but could involve, for example, slight crack deflections due to the inhomogeneities present in the material (Dong et al., 2024).

### 4.4. Decreases in toughness observed at low-to-moderate concentrations (objective 2c)

For all three measures of toughness, decreases were observed up to 2.5 wt% from the initial peak values at low concentrations (0.25 wt% for tensile, 0.5 wt% for impact and fatigue). In the imaging results, these decreases coincided with two features of note: (i) the formation of noticeable intergranular voids in strained SEM images (e.g., Fig. 15b), and (ii) the transition from criss-cross fracture surface features to more spheroid features, indicative of a transition from transgranular to intergranular fracture modes (Figs. 10 and 11).

Regarding (i), clear decreases in toughness and changes in fracture mode above ~0.25 to 0.5 wt% may be associated with the formation of intergranular regions containing just enough carbon black to allow for the formation of void-like intergranular stress concentrators. Li et al. discussed that such stress concentrators in UHMWPE composites can result in expanding crazes which then accumulate under continuous loading (Li et al., 2017). This then could lead to initiation of a sharp tensile crack that can propagate readily across the material in the case of a tensile sample, or through a local region at the crack front in the case of fatigue or impact samples. Similarly, recent work (Inverardi et al., 2025) studied materials for which partial granule coating appeared in micrographs for several of their material treatments (thus, somewhat analogous to the behavior below 2.5 wt% in our results). They found that composites with a greater extent of brittle intergranular stress-concentration regions tended to have degraded tensile and impact properties compared to materials with lesser extent of such regions. Thus, the significant dip in mechanical properties from low to moderate concentration in this study is to be expected, as the magnitude of intergranular stress concentration becomes larger with increasing extent of filler-rich intergranular domains (albeit, ones that are thoroughly transected with polymer fibrils). However, once complete granule coating occurs, the physical deformation behavior appears to change, leading to a flattening of the tensile and impact toughness curves across moderate filler concentrations.

Regarding (ii), the transition to intergranular fracture should coincide with higher filler concentration and weaker granule boundaries, as the filler will partially hinder diffusion of polymer chains into adjacent granules (Senatov et al., 2014). While these weaker granule boundaries may lead to crack tip diversion and a more tortuous fracture pathway, that toughening effect may not be large enough to overcome the concomitant loss in toughness due to the limited polymer material available for energy absorption in the intergranular regions.

While these decreases in the mechanical properties at low-to-moderate concentrations are notable, the drops are still relatively small compared to what some others have noted when approaching the onset of complete granule coating. Senatov et al. found that the strain at break of their UHMWPE composites decreased significantly around the onset of complete granule coating, and they attributed this to the poor ability for bonding to occur between polymer granules across the filler boundary in their nanocomposites (e.g., tungsten nanoparticles in UHMWPE) (Senatov et al., 2014). On the other hand, SEM images shown in this work demonstrate extensive integration of UHMWPE into the intergranular regions. This is perhaps similar to the effect in play for submicron drug particle loading in UHMWPE, which maintained favorable impact toughness across a range of filler loadings, attributed to good fusion of the polymer chains across granule boundaries (Asik et al., 2025). The fibrous nature of the intergranular polymer might be leading to better stress transfer through adjacent granules or obstruction of crack propagation (Yesgat and Kitey, 2016), similar to the crack tip bridging effects noted in the discussion of fatigue results. This effect might be limiting the decreases in mechanical properties experienced in these carbon black-UHMWPE composites at low-to-moderate concentrations in addition to the higher concentrations discussed earlier.

### 4.5. Limitations

While this work sought to quantify the behavior of UHMWPE composites and understand the potential structural reasons for that behavior, it did not seek to prove structure-property relationship causality, making this one of the primary limitations of this study. A related limitation is that the extent to which the observed microstructural phenomena contributed to the observed macroscopic properties was not determined. For example, while stress distribution at the crack tip was inferred based on observations in SEM, it was not directly observed or quantified, and the strength of its effect relative to other effects was not determined.

This work was also limited in the sense that not all microstructural features of the material were considered in the analysis. Crystallinity is an important contributor to UHMWPE mechanical properties, but remained outside of the scope of this analysis. While we briefly discussed the role of plastic deformation at the granule scale and intergranular scale, a distinction between plastic deformation in amorphous versus crystalline domains was not investigated. Published work has shown that crystallinity could be expected to have an effect on material toughness (Oral et al., 2009), but mixed results have been obtained concerning the effect of fillers on crystallinity in UHMWPE composites (Duraccio et al., 2019; Qu et al., 2019). Furthermore, interactions between UHMWPE polymer chains and carbon filler particles can be expected, and have already been shown to produce changes in crystallite size (Mindivan and Dere, 2024). Thus, future studies should be undertaken to understand the effect of crystallinity or other polymeric microstructural factors, such as residual stresses, on the deformation behavior in these composites. Similarly, future studies should investigate the effect of certain processing variables (e.g. consolidation time) on the results, as some differences in fatigue fracture surface appearance were observed for samples taken from the center of the billets. Accordingly, a limitation of this study is that the effect of the composites’ thermal conductivity was not considered, but it could influence the extent of polymer chain diffusion across granule boundaries.

A limitation of the mechanical results presented here is that they can only be compared to studies utilizing the same sample geometries. The tensile samples were modified (thinner) ASTM Type V samples, which allow for comparison to previous studies and retrieval work completed in the literature using the modified form. Thinner tensile samples of UHMWPE are known to reach higher levels of ultimate tensile stress while maintaining similar ductility, and thus will tend to have higher tensile toughness than thicker samples (Morrison and Jani, 2009). Thus, these results cannot be directly compared to work using thicker samples. In a similar way, small fatigue samples with geometry defined by (Baker et al., 2003) have a different ratio of plane stress to plane strain than some of the larger, thicker samples also used in the literature, so the results cannot be directly compared.

The defect-tolerant fatigue testing approach used in this study is limited by making certain linear elastic fracture mechanics (LEFM) assumptions, which do not apply perfectly to materials with high crack tip plasticity such as UHMWPE and its composites (Furmanski and Pruitt, 2007; Sirimamilla et al., 2013). However, this approach has been used to perform many comparative studies on UHMWPE materials with similar bulk properties to the materials studied here, so the comparisons made here among composites of UHMWPE are believed to be informative as plastic zone size should be similar to historical UHMWPE materials studied under LEFM.

Finally, a broad limitation of this study is that the three toughness measures, though representative of a wide array of potential failure modes, leave other areas left to study to fully understand these materials’ mechanical response to clinically-relevant complex states of stress (Kennedy et al., 2000). Ultimately, component-level testing and careful analysis of materials retrieved after time in vivo will be necessary to determine the mechanical viability of these materials for long-term use in the body.

## 5. Conclusion

One of the principal findings of this study was that the distinct phase segregation of UHMWPE composites causes them to behave differently than crosslinked UHMWPE materials studied extensively in the literature. In this work, tensile and impact toughness followed similar trends to each other, while the fatigue crack propagation resistance deviated from those measures at higher concentration. This study demonstrates that for these phase-segregated materials, tensile or impact properties cannot necessarily be used as a proxy for the fatigue properties of the materials. We also observed that certain microstructural phenomena corresponded to macroscopic property changes. These phenomena included the transition from transgranular to intergranular fracture, the onset of complete granule coating, and the formation of intergranular voids. The results presented here could influence understanding of how similar solid-state composites behave across a range of relevant loading scenarios and concentrations and could aid in the design of future forms of particulate-loaded UHMWPE composites. For example, this work suggests that it may be possible to attain higher levels of fatigue CPR with higher levels of nanofiller loading, if a sufficient level of intergranular polymer diffusion can be obtained across wide granule boundary regions. This study lays the foundation for continued work to develop an understanding of phase-segregated polymer composites’ mechanical behavior in response to various microstructural phenomena and will enable better tuning of that microstructure towards applications of interest. Finally, while applications were not the subject of this study, the results herein demonstrate promise for use of these materials in high-load scenarios, whether they be in arthroplasty or any other field in which the unique properties of UHMWPE composites prove beneficial.

## Supplementary Material

1

## Figures and Tables

**Table 1:** Materials and mechanical test methods used in this study.

Test Name	Materials	Method
Tensile testing	0, 0.25, 0.5, 0.75, 1, 2.5, 5, 7.5, 10 wt% PCB in GUR 1020 UHMWPE N = 15 each	ASTM D638 (using 200 μm thick sections)
Impact testing	0, 0.5, 1, 2.5, 5, 10 wt% PCB in GUR 1020 UHMWPE N = 5 each	Double-Notched Izod (DNI) Testing ASTM F648, D256
Fatigue testing	0, 0.5, 1, 2.5, 5, 10 wt% PCB in GUR 1020 UHMWPE N = 5 each	*Sample geometry:* Baker et al., (2003); *Testing parameters:* Atwood et al., 2011, adapted from ASTM E647 for use with UHMWPE

**Table 2:** Transformed da/dN curve parameters for linear regressions, used to compute 95% CIs about the data and determine the 10^−5^ and 10^−4^ intercept values representing the left-right shift of the da/dN curves.

*Value* ± *SE*	Neat	0.5 wt%	1 wt%	2.5 wt%	5 wt%	10 wt%
**Slope**	9.28 ± 0.33	8.36 ± 0.43	8.91 ± 0.53	9.37 ± 0.86	10.94 ± 0.94	10.69 ± 0.83
**Y-int.**	−6.68 ± 0.08	−6.55 ± 0.11	−6.40 ± 0.13	−6.19 ± 0.19	−6.59 ± 0.20	−6.70 ± 0.19

## Data Availability

Data will be made available on request.

## Appendix A. Supplementary data

Supplementary data to this article can be found online at https://doi.org/10.1016/j.jmbbm.2025.107136.
