# Supplementary material for: Toughness measures in solid-state composites of ultra-high molecular weight polyethylene
Source: J Mech Behav Biomed Mater. Author manuscript; Available in PMC 2026 Sep 18. (PMC13587155; doi:10.1016/j.jmbbm.2025.107136)
Supplement: Supplementary file 1 [file NIHMS2180172-supplement-1.docx]

***Supplemental Material For “Toughness Measures in Solid-State Composites of Ultra-High Molecular Weight Polyethylene” by Solberg, Tsukrov, and Van Citters***

**S1: Tensile Toughness Statistical Results – Comprehensive**

Table S1: Statistical results for tensile toughness - Dunnett’s T3 Multiple Comparisons test following Brown-Forsythe ANOVA, as compiled in GraphPad Prism. The threshold for statistical significance was p=0.05.

| **Dunnett's T3 multiple comparisons test** | **Below threshold?** | **Summary** | **Adjusted P Value** | **Mean 1 (MPa)** | **Mean 2 (MPa)** |
| --- | --- | --- | --- | --- | --- |
| Neat vs. 0.25wt% | No | ns | 0.9940 | 149.0 | 156.2 |
| Neat vs. 0.5wt% | No | ns | 0.4173 | 149.0 | 133.6 |
| Neat vs. 0.75wt% | Yes | ** | 0.0057 | 149.0 | 121.9 |
| Neat vs. 1.0wt% | Yes | * | 0.0159 | 149.0 | 126.4 |
| Neat vs. 2.5wt% | Yes | ** | 0.0024 | 149.0 | 120.9 |
| Neat vs. 5.0wt% | Yes | ** | 0.0016 | 149.0 | 120.5 |
| Neat vs. 7.5wt% | Yes | **** | <0.0001 | 149.0 | 108.6 |
| Neat vs. 10wt% | Yes | *** | 0.0001 | 149.0 | 113.8 |
| 0.25wt% vs. 0.5wt% | Yes | *** | 0.0005 | 156.2 | 133.6 |
| 0.25wt% vs. 0.75wt% | Yes | **** | <0.0001 | 156.2 | 121.9 |
| 0.25wt% vs. 1.0wt% | Yes | **** | <0.0001 | 156.2 | 126.4 |
| 0.25wt% vs. 2.5wt% | Yes | **** | <0.0001 | 156.2 | 120.9 |
| 0.25wt% vs. 5.0wt% | Yes | **** | <0.0001 | 156.2 | 120.5 |
| 0.25wt% vs. 7.5wt% | Yes | **** | <0.0001 | 156.2 | 108.6 |
| 0.25wt% vs. 10wt% | Yes | **** | <0.0001 | 156.2 | 113.8 |
| 0.5wt% vs. 0.75wt% | No | ns | 0.5401 | 133.6 | 121.9 |
| 0.5wt% vs. 1.0wt% | No | ns | 0.8956 | 133.6 | 126.4 |
| 0.5wt% vs. 2.5wt% | No | ns | 0.2751 | 133.6 | 120.9 |
| 0.5wt% vs. 5.0wt% | No | ns | 0.0962 | 133.6 | 120.5 |
| 0.5wt% vs. 7.5wt% | Yes | ** | 0.0014 | 133.6 | 108.6 |
| 0.5wt% vs. 10wt% | Yes | ** | 0.0030 | 133.6 | 113.8 |
| 0.75wt% vs. 1.0wt% | No | ns | 0.9995 | 121.9 | 126.4 |
| 0.75wt% vs. 2.5wt% | No | ns | >0.9999 | 121.9 | 120.9 |
| 0.75wt% vs. 5.0wt% | No | ns | >0.9999 | 121.9 | 120.5 |
| 0.75wt% vs. 7.5wt% | No | ns | 0.3629 | 121.9 | 108.6 |
| 0.75wt% vs. 10wt% | No | ns | 0.8354 | 121.9 | 113.8 |
| 1.0wt% vs. 2.5wt% | No | ns | 0.9683 | 126.4 | 120.9 |
| 1.0wt% vs. 5.0wt% | No | ns | 0.5557 | 126.4 | 120.5 |
| 1.0wt% vs. 7.5wt% | Yes | * | 0.0107 | 126.4 | 108.6 |
| 1.0wt% vs. 10wt% | Yes | ** | 0.0066 | 126.4 | 113.8 |
| 2.5wt% vs. 5.0wt% | No | ns | >0.9999 | 120.9 | 120.5 |
| 2.5wt% vs. 7.5wt% | No | ns | 0.3715 | 120.9 | 108.6 |
| 2.5wt% vs. 10wt% | No | ns | 0.8564 | 120.9 | 113.8 |
| 5.0wt% vs. 7.5wt% | No | ns | 0.2250 | 120.5 | 108.6 |
| 5.0wt% vs. 10wt% | No | ns | 0.5428 | 120.5 | 113.8 |
| 7.5wt% vs. 10wt% | No | ns | 0.9988 | 108.6 | 113.8 |

S2: Impact Toughness Statistical Results – Comprehensive

Table S2: Statistical results for impact toughness - Dunnett’s T3 Multiple Comparisons test following Brown-Forsythe ANOVA, as compiled in GraphPad Prism. The threshold for statistical significance was p=0.05.

| **Dunnett's T3 multiple comparisons test** | **Below threshold?** | **Summary** | **Adjusted P Value** | **Mean 1 (kJ/m^2^)** | **Mean 2 (kJ/m^2^)** |
| --- | --- | --- | --- | --- | --- |
| Neat vs. 0.5wt% | Yes | ** | 0.0026 | 135.8 | 141.9 |
| Neat vs. 1.0wt% | No | ns | 0.0676 | 135.8 | 130.1 |
| Neat vs. 2.5wt% | Yes | ** | 0.0017 | 135.8 | 114.8 |
| Neat vs. 5wt% | Yes | **** | <0.0001 | 135.8 | 114.5 |
| Neat vs. 10wt% | Yes | **** | <0.0001 | 135.8 | 100.5 |
| 0.5wt% vs. 1.0wt% | Yes | ** | 0.0017 | 141.9 | 130.1 |
| 0.5wt% vs. 2.5wt% | Yes | *** | 0.0006 | 141.9 | 114.8 |
| 0.5wt% vs. 5wt% | Yes | **** | <0.0001 | 141.9 | 114.5 |
| 0.5wt% vs. 10wt% | Yes | **** | <0.0001 | 141.9 | 100.5 |
| 1.0wt% vs. 2.5wt% | Yes | ** | 0.0040 | 130.1 | 114.8 |
| 1.0wt% vs. 5wt% | Yes | *** | 0.0001 | 130.1 | 114.5 |
| 1.0wt% vs. 10wt% | Yes | **** | <0.0001 | 130.1 | 100.5 |
| 2.5wt% vs. 5wt% | No | ns | >0.9999 | 114.8 | 114.5 |
| 2.5wt% vs. 10wt% | Yes | ** | 0.0089 | 114.8 | 100.5 |
| 5wt% vs. 10wt% | Yes | *** | 0.0002 | 114.5 | 100.5 |

**S3: Determination of Statistical Significance of Fatigue Results**

As described in the main text, to determine the right-left shift of the respective da/dN curves, the data were log transformed, fitted with a linear least-squares regression line, and the log(∆K) values corresponding to log(da/dN) values of -5 and -4 were found. These measures of fatigue crack propagation resistance (CPR) were given by:

$$I={10}^{x}={10}^{\left( -5-a \right)/b}$$

which is simply the back-transformation of the linear regression of the transformed data in the form $y=bx+a$, where a is the y-intercept of the fit of the log-transformed values, b is slope, and y = -5. An alternative approach would have been to do a nonlinear fit of the original data on a log-log plot and report the resulting slope and intercept. These results may end up being similar to those found with the transformed data, but the data from higher y values would be weighted much more heavily than those with lower y values as this method minimizes the sum of squares of the difference between the actual Y value and the Y value predicted by the fitted curve^1^. Equal weighting of all points on the curve is desired, hence the use of transformed data to find a linear fit.

Comparisons between curves were made in the log-transformed regime using the 95% confidence intervals (CIs) constructed in GraphPad Prism with the following equation:

$$\hat{y}_{c}=\hat{y}\pm t_{n-2,a/2}\sqrt{\frac{\sum_{i=1}^{n} \left( y_{i}-\hat{y} \right)^{2}}{n-2}}*\sqrt{\frac{1}{n}+\frac{\left( x-\bar{x} \right)^{2}}{\sum_{i=1}^{n} \left( x_{i}-\bar{x} \right)^{2}}} (Equation S1)$$

The meaning of these intervals is the following: we are 95% confident that the true linear regression falls within the 95% confidence interval; since the 10^-5^ intercept is taken from this predictor (regression line), we are likewise 95% confident that the true 10^-5^ intercept value falls within the CI. The same meaning holds true for the 10^-4^ intercept. Any overlap in CIs at the transformed crack growth rate of interest (e.g. -5) was thus indicative of statistical non-significance at the α=0.05 level while lack of overlap indicated significance.

With five samples for each materials treatment, and an average of about 10 data points per sample, each material treatment had about 50 observations (range: 45 to 55). Since each of these is an observation from the same material treatment, regardless of sample, analysis was performed on all the data points from each material treatment, combined. With sample size sufficiently high (>30 or 40 per Ghasemi and Zahedias^2^), any violations of normality are not a significant concern and parametric procedures can be used even if the data are not normally distributed^3^.

Using these data and the method outlined above, Figure 6 in the main text was produced. Regarding the 95% confidence intervals, note that the 95% CI of the predictor line was used to determine the intercept values, not the 95% CIs of the slopes and y intercepts provided in Table 2 of the main text. These confidence intervals of the predictor lines are duplicated in this supplemental in Figure S1. Results of 95% CI overlap comparisons to determine statistical significance of the pairings at the two crack growth levels are included in Table 1.

Figure S1 (duplicated from Figure 6 in the main text): log_10_-transformed fatigue results with 95% confidence intervals (CIs) indicated by shaded regions

Table S3: Comparisons of 95% confidence intervals of all composite material treatments for fatigue crack propagation resistance. Overlap of 95% CIs at each log(da/dN) level indicated lack of statistical significance, while no overlap indicated statistical significance at that crack growth rate. Differences between the 10^-5^ and the 10^-4^ intercept results were minimal but are bolded in the table.

| ***Pairing:*** | **10^-5^ da/dN Intercepts: Significant Pair?** | **10^-4^ da/dN Intercepts:**  **Significant Pair?** |
| --- | --- | --- |
| Neat vs. 0.5 wt. % | **No** | **Yes** |
| Neat vs. 1 wt. % | Yes | Yes |
| Neat vs. 2.5 wt. % | Yes | Yes |
| Neat vs. 5 wt. % | Yes | Yes |
| Neat vs. 10 wt. % | Yes | Yes |
| 0.5 wt. % vs. 1 wt. % | Yes | Yes |
| 0.5 wt. % vs. 2.5 wt. % | Yes | Yes |
| 0.5 wt. % vs. 5 wt. % | Yes | Yes |
| 0.5 wt. % vs. 10 wt. % | Yes | Yes |
| 1 wt. % vs. 2.5 wt. % | Yes | Yes |
| 1 wt. % vs. 5 wt. % | **No** | **Yes** |
| 1 wt. % vs. 10 wt. % | No | No |
| 2.5 wt. % vs. 5 wt. % | No | No |
| 2.5 wt. % vs. 10 wt. % | Yes | Yes |
| 5 wt. % vs. 10 wt. % | No | No |

**S4: Summary of Results**

Table S4 summarizes mechanical property results obtained in this paper.

Table S4: Summary of mechanical properties presented in the paper (listed as value ± SD, when applicable)

| **Concentration**  (wt.% CB in UHMWPE) | **Tensile Toughness**  (MPa)  n=15 | **UTS**  (MPa)  n=15 | **Strain at Break**  (%)  n=15 | **Impact Toughness**  (kJ/m^2^)  n=5 | **Fatigue CPR**  **∆K at da/dN=10^-5^ or 10^-4^ mm/cy**  (MPa√m)  n=5 specimens providing 45-55 data pts per concentration | |
| --- | --- | --- | --- | --- | --- | --- |
|  | | | | | *10^-5^* | *10^-4^* |
| 0 | 149.0 ± 19.3 | 65.4 ± 8.3 | 450.6 ± 30.2 | 135.8 ± 1.2 | 1.52 | 1.94 |
| 0.25 | 156.2 ± 8.2 | 67.5 ± 3.8 | 459.3 ± 12.0 | - | - | - |
| 0.5 | 133.6 ± 13.8 | 58.0 ± 6.1 | 422.0 ± 24.8 | 141.9 ± 1.6 | 1.53 | 2.02 |
| 0.75 | 121.9 ± 13.8 | 52.6 ± 6.2 | 407.8 ± 29.9 | - | - | - |
| 1 | 126.4 ± 7.3 | 55.2 ± 3.2 | 413.0 ± 13.0 | 130.1 ± 2.7 | 1.43 | 1.86 |
| 2.5 | 120.9 ± 11.6 | 52.0 ± 4.9 | 398.1 ± 24.7 | 114.8 ± 4.6 | 1.34 | 1.71 |
| 5 | 120.5 ± 6.8 | 52.1 ± 3.0 | 394.5 ± 12.7 | 114.5 ± 2.2 | 1.40 | 1.72 |
| 7.5 | 108.6 ± 14.4 | 46.6 ± 5.5 | 372.5 ± 35.2 | - | - | - |
| 10 | 113.8 ± 8.6 | 47.7 ± 3.6 | 384.5 ± 17.5 | 100.5 ± 2.5 | 1.44 | 1.79 |

References for Supplemental Materials:

1.	GraphPad Prism 10 Curve Fitting Guide - Equation: Fitting a straight line on a semi-log or log-log graph. Accessed July 16, 2024. https://www.graphpad.com/guides/prism/latest/curve-fitting/reg_fitting_lines_to_semilog.htm

2.	Ghasemi A, Zahediasl S. Normality Tests for Statistical Analysis: A Guide for Non-Statisticians. *Int J Endocrinol Metab*. 2012;10(2):486-489. doi:10.5812/ijem.3505

3.	Elliott AC, Woodward WA. *Statistical Analysis Quick Reference Guidebook: With SPSS Examples*. SAGE; 2007.
